# The E3 ubiquitin ligase HUWE1 is required for KRAS-induced lung cancer

**DOI:** 10.1038/s41419-026-08672-7

**Published:** 2026-04-07

**Authors:** J. Searle, M. Menotti, W. J. McDaid, M. J. Baker, A. Chaturvedi, L. Ginn, H. Reed, M. Carter, F. Blackhall, C. R. Lindsay, A. Malliri

**Affiliations:** 1 Cell Signalling Group, Cancer Research UK Manchester Institute, The University of Manchester, Manchester, UK; 2 Cancer Research UK Lung Cancer Centre of Excellence, The University of Manchester, Manchester, UK; 3 Division of Cancer Sciences, School of Medical Sciences, Faculty of Biology, Medicine and Health, The University of Manchester, Manchester, UK; 4 The Christie NHS Foundation Trust, Manchester, UK; 5 Present Address: Lungs for Living Research Centre, UCL Respiratory, Division of Medicine, University College London (UCL), London, UK

**Keywords:** Non-small-cell lung cancer, Ubiquitylation

## Abstract

The E3 ubiquitin ligase HUWE1 modifies a diverse network of substrate proteins by ubiquitination, through which it regulates various intracellular processes and contributes to both oncogenic and tumour suppressor mechanisms in different cancer contexts. Here, by analysing human lung adenocarcinoma (LUAD) patient samples, we reveal that HUWE1 protein expression is commonly upregulated in LUAD tumours compared to normal adjacent lung tissue and that this increase is associated with tumour stage. Using multiple, independent murine models of LUAD initiation and growth, we identify that *Huwe1* is essential for mutant *Kras*-induced lung tumour development and reveal a novel, p53-independent requirement for *Huwe1* in LUAD. Mechanistically, we demonstrate induction of senescence following HUWE1 depletion - characterised by impaired proliferation, an atypical cell cycle distribution, emergence of morphologically abnormal enlarged cells, increased β-galactosidase activity, and transcriptional reprogramming associated with inflammatory senescence-associated secretory phenotype (SASP) signalling and NFκB activation. Together, these data highlight a crucial role for HUWE1 in mutant *Kras*-induced LUAD tumorigenesis and in the continued growth and proliferation of established LUAD cells, confirming HUWE1 as a rational therapeutic target for LUAD.

## Introduction

Ubiquitination is a mechanism of post-translational modification in which the small 8.5 kDa protein ubiquitin is appended to intracellular substrate proteins through a three-step mechanism culminating in the activity of an E3 ubiquitin ligase [1]. This may result in proteasomal degradation of the substrate protein or other non-degradative changes in protein fate, including alterations in subcellular localisation, scaffolding functions, or in the activity of transcription factors and kinases [2]. The fate of a ubiquitinated substrate is typically related to the specific ubiquitin modification added, which ranges from monoubiquitination to the addition of extensive chains of polyubiquitin – which can be joined by one of 8 linkage types and may exhibit branching [3, 4].

HUWE1 is an HECT-family E3 ubiquitin ligase that ubiquitinates a broad range of substrate proteins, through which it can regulate proliferation, cell survival, migration, transcription factor activity, DNA damage repair, and maintenance of proteostasis [5, 6]. Unlike many other E3 ligases, HUWE1 can append a variety of different ubiquitin modifications to substrates, including monoubiquitination and a diverse range of branched and unbranched polyubiquitin linkages [6]. Studies have revealed a critical role for HUWE1 in embryonic and neurological development, with germline knockout of *Huwe1* resulting in murine embryonic lethality [7–9], and loss-of-function mutations in *HUWE1* resulting in human intellectual disability and developmental disorders [10–12]. In cancer, both tumour suppressive and oncogenic functions of HUWE1-mediated ubiquitination have been identified in different cancer contexts [5]. These opposing functions arise through the ubiquitination and regulation of various substrates, including p53 [8,13–15], c-Myc [16, 17], Miz1 [18, 19], Mcl1 [17,20–22], and BRCA1 [23]. HUWE1 expression is elevated in several cancer types, including colon cancer, multiple myeloma, breast cancer, and lung cancer [16, 24, 25].

Lung Adenocarcinoma (LUAD) is the most common histological subtype of Non-Small Cell Lung Cancer, accounting for ~40% of all lung cancer cases [26], and is commonly diagnosed as advanced stage, metastatic disease for which prognosis is poor. Treatment of advanced disease typically involves targeted inhibition of frequently mutated oncoproteins such as KRAS and EGFR, alongside the use of chemotherapy and immunotherapy. However, the onset of acquired resistance is common, and survival rates for patients with advanced-stage disease remain poor (1-year overall survival for Stage IV LUAD: 21%) [27]. Novel therapeutic strategies are necessary for improved monotherapy and combination approaches, with the ubiquitin system representing a rational target. Several E3 ubiquitin ligases are dysregulated in LUAD [28], and inhibition of the ubiquitin-proteasome system has previously shown clinical promise - the proteasome inhibitor bortezomib yielded moderate anti-tumour responses in some LUAD patients [29–31], with exceptional responses observed in patients harbouring a *KRAS* G12D mutation and invasive mucinous adenocarcinoma (IMA) histology [32]. Additionally, the only hit to arise across multiple independent synthetic lethality screens in *RAS* mutant cancers is the proteasome, demonstrating the importance of the ubiquitin-proteasome system in *KRAS* mutant LUAD [33]. However, FDA-approved proteasome inhibitors suffer severe dose-limiting toxicity due to inhibition of proteasome function in normal, healthy tissue [30]. Hence, a more tolerable, efficacious approach centres on inhibiting the ubiquitin-proteasome system upstream by targeting E3 ubiquitin ligases upon which LUAD tumours are uniquely reliant.

To date, few studies have interrogated the functional importance of HUWE1 in LUAD tumorigenesis and malignant progression. We have previously shown that HUWE1 ubiquitinates the RAC1 activator TIAM1 at cell-cell junctions in LUAD cells following HGF stimulation, resulting in the proteasomal degradation of TIAM1, junctional disassembly, and increased cell migration [34]. Yang and colleagues have also identified that HUWE1 ubiquitinates p53, resulting in its proteasomal degradation and increased NSCLC cell survival [13]. In this study, we sought to interrogate whether HUWE1 is essential for the initiation and growth of *Kras*-mutant LUAD tumours and establish if it represents a rational therapeutic target.

Herein, we identify that HUWE1 protein expression is upregulated in LUAD compared to matched normal tissue and show an essential oncogenic requirement for *Huwe1* in mutant *Kras*-induced LUAD, independent of its known regulation of p53. We further identify that human LUAD cells devoid of HUWE1 exhibit impaired proliferation leading to the induction of senescence, and that established subcutaneous LUAD tumour xenografts suffer reduced growth following HUWE1 loss. These findings determine that HUWE1 is required for LUAD tumorigenesis and continued tumour cell proliferation and poses a rational target for therapeutic inhibition to impair LUAD tumour growth and improve patient survival.

## Results

### HUWE1 expression is upregulated in LUAD patients

**Fig. 1:**
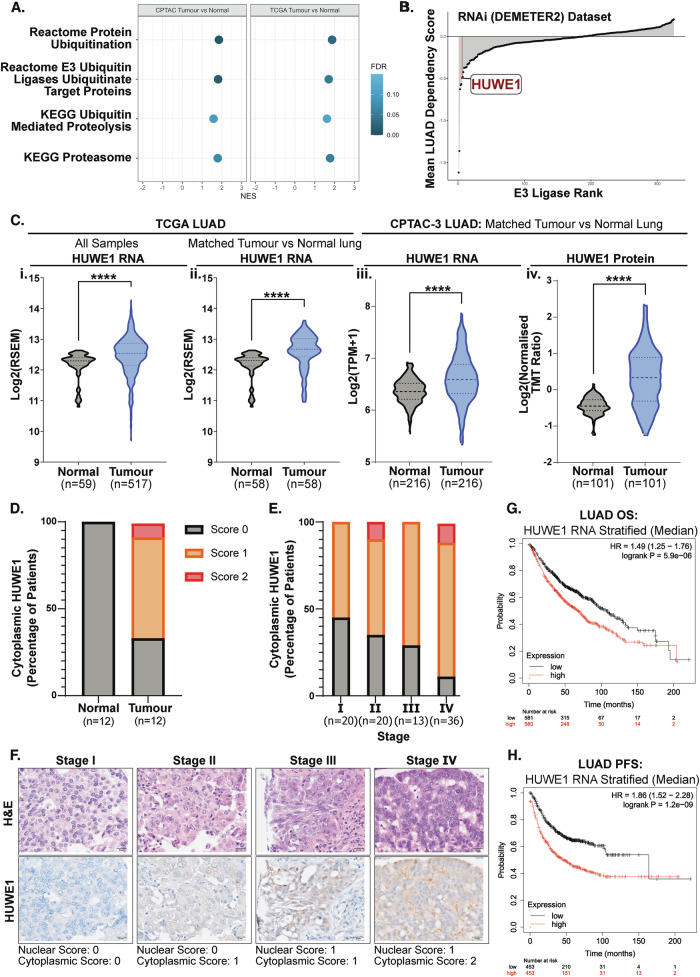
HUWE1 expression is upregulated in LUAD compared to the normal lung. **A** Significantly enriched Reactome and KEGG gene sets associated with ubiquitination and proteasomal degradation from gene set enrichment analysis (GSEA) of matched LUAD tumour versus normal adjacent lung samples from CPTAC-3-LUAD (*n* = 216) and TCGA-LUAD (*n* = 58) bulk transcriptomic data. NES denotes normalised enrichment score. FDR denotes the false discovery rate q-value. **B** E3 ubiquitin ligases ranked by mean dependency score for LUAD cell lines with HUWE1 highlighted, from RNAi (DEMETER2) dependency data from DepMap (Broad Institute). **C** HUWE1 RNA expression in the TCGA-LUAD dataset comparing (i) all LUAD tumour (*n* = 517) and normal adjacent lung (*n* = 59) samples, (ii) matched pairs of LUAD and normal lung samples (*n* = 58 pairs). (iii) HUWE1 RNA and (iv) protein expression in CPTAC-3-LUAD dataset comparing matched pairs of LUAD tumour and normal adjacent lung samples (*n* = 216 pairs, 101 pairs respectively). Mann-Whitney unpaired test in (i), Wilcoxon signed rank paired test in (ii), paired T-test in (iii) and (iv). **D** Assessment of cytoplasmic HUWE1 IHC staining by tumour stage in *n* = 12 patients with matched normal tissue sections. **E** Assessment of cytoplasmic HUWE1 IHC staining by tumour stage in LUAD cohort of *n* = 89 patients. **F** Representative H&E-stained and HUWE1-stained serial sections of LUAD tumour tissue by tumour stage, with annotated stain intensity scores. Scale bar 20 μm. **G** Kaplan-Meier analysis of LUAD patient overall survival (OS) stratified by median HUWE1 RNA expression. Generated using the KMplotter tool for cohort combining LUAD patients from multiple independent studies, *n* = 1161 patients. **H** As in G for progression-free survival (PFS), *n* = 906 patients.

As HUWE1 has been implicated in several cancers, we started by examining the expression of HUWE1 and other components of ubiquitin signalling in LUAD patient samples. Gene set enrichment analysis (GSEA) of bulk transcriptomic data from multiple, independent LUAD patient datasets revealed that pathways and gene sets associated with ubiquitination are transcriptionally upregulated in LUAD compared to normal adjacent lung tissue (Fig. 1A, S1A). Assessment of CRISPR and RNAi essentiality screening data from the Dependency Mapping project (DepMap, Broad Institute [35]) revealed that in LUAD cancer cell lines, HUWE1 is amongst the topmost essential E3 ubiquitin ligases for cell viability, ranking in the top 4% and 2% of essential E3s, respectively **(**Fig. 1B, S1B**)**. Together, these analyses suggest that ubiquitination machinery is upregulated in LUAD, and that the E3 ubiquitin ligase HUWE1 may play a central role in the continued growth and survival of LUAD cells.

Subsequent assessment of HUWE1 mRNA and protein expression in published LUAD patient datasets revealed a significant upregulation in LUAD tumour tissue compared to matched normal adjacent lung tissue (Fig. 1Ci–iv). To corroborate these findings in an independent cohort, we optimised HUWE1 immunohistochemistry (IHC) in human lung samples (Fig. S2A, B) and stained LUAD tumours from 89 patients – for 12 of whom we also stained normal adjacent lung tissue. Assessment of HUWE1 stain intensity by a histopathologist (Fig. S2C) revealed that cytoplasmic HUWE1 expression was increased in LUAD tumour tissue compared to matched normal lung tissue (HUWE1 positivity: normal lung 0% vs LUAD tumour 67%) (Fig. 1D). Moreover, across the cohort of 89 LUAD patients we observed an increase in cytoplasmic HUWE1 positivity by tumour stage, ranging from 55% HUWE1 positivity at stage I, to 88% at stage IV (Fig. 1E, F). Whilst higher HUWE1 expression did not correlate with patient survival in our dataset (Fig. S2D), analysis of a considerably larger cohort of *n* = 1161 LUAD patients combined from several independent studies revealed that high HUWE1 mRNA expression is significantly associated with poorer overall survival (OS) and progression-free survival (PFS) (Fig. 1G, H). Nuclear HUWE1 expression also showed an increase with histological tumour stage (Fig. S3 A–C). Together, these data indicate that HUWE1 expression is elevated in LUAD compared to the normal lung, is more commonly increased in advanced-stage disease, and high expression is prognostic of poorer patient survival. From these findings, we hypothesized that elevated HUWE1 expression in LUAD may suggest an important functional role for HUWE1 in lung tumorigenesis and malignant progression.

### *Huwe1* genetic depletion impairs mutant *Kras*-induced LUAD development in vivo

To assess the functional contribution of HUWE1 in LUAD tumorigenesis, we employed a variety of murine models that incorporate mutation of *Kras*, which is present in ~25% of LUAD patients[36], to induce lung tumour formation.

**Fig. 2:**
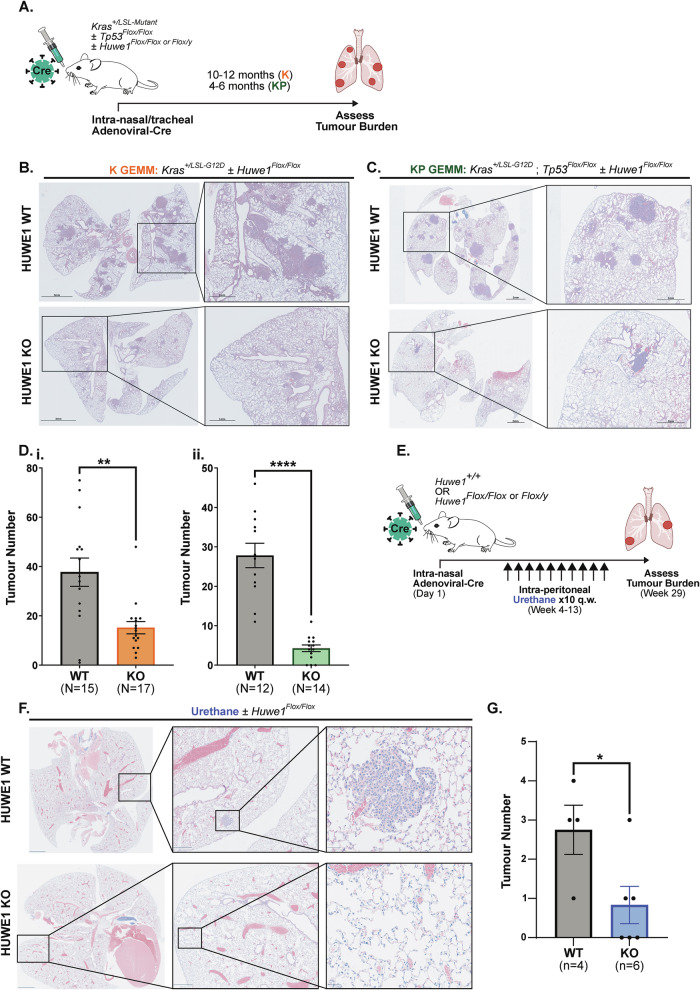
*Huwe1* genetic depletion impairs mutant *Kras*-induced LUAD development in vivo. **A** Experimental timeline for Cre-lox inducible models of *Kras*-induced lung tumorigenesis. K GEMM: *Kras*^*+/LSL-G12D*^; KP GEMM: *Kras*^*+/LSL-G12D*^, *Tp53*^*Flox/Flox*^. **B** Representative H&E-stained lung sections from *Huwe1*^*+/+*^ wild type (WT) and *Huwe1*^*Flox/Flox or Flox/y*^ knockout (KO) mice in K GEMM. Scale bar: 3 mm; inset scale bar:1 mm. **C** As in B, for KP GEMM. Scale bar: 2 mm; inset scale bar:1 mm. **D** Quantification of tumour number in *Huwe1* WT and KO mice in K GEMM **(i)** (*n* = 15 *Huwe1* WT, *n* = 17 *Huwe1* KO) and KP GEMM **(ii)** (*n* = 12 *Huwe1* WT, *n* = 14 *Huwe1* KO). Shown as mean± SEM, Mann-Whitney non-parametric test. **E** Experimental timeline for urethane carcinogenesis model of *Kras*-induced lung tumorigenesis. **F** Representative H&E-stained lung sections from *Huwe1*^+/+^ wild type (WT) and *Huwe1*^*Flox/Flox or Flox/y*^ knockout (KO) mice in the urethane tumorigenesis model. Scale bar: 2 mm; first inset scale bar: 500 μm; second inset scale bar: 50 μm. **G** Quantification of tumour number in *Huwe1* WT and KO mice from urethane tumorigenesis study (*n* = 4 *Huwe1* WT, *n* = 6 *Huwe1* KO). Shown as mean ± SEM, unpaired T-test with Welch’s correction.

Firstly, we initiated mutant *Kras*-induced lung tumorigenesis by utilising a *Kras*^*+/Lox-STOP-Lox-G12D*^ genetically engineered mouse model (K GEMM). Upon intranasal/intratracheal administration of Adenoviral-CMV-Cre (Ad-Cre), recombination of LoxP sites resulted in the excision of the STOP cassette and expression of mutant *Kras* G12D in the lung. This resulted in the development of distal lung tumours typically comprising adenomas and adenocarcinomas (Fig. 2A). By introducing a *Huwe1*^*Flox/Flox or Flox/y*^ allele we were able to assess the impact of conditional *Huwe1* depletion on *Kras*-induced lung tumorigenesis, whereby Cre administration resulted in the concomitant expression of mutant *Kras* G12D and knockout of *Huwe1* via excision of essential exons 80-82 (Fig S4A). Interestingly, mice with *Huwe1* depletion (KO) developed significantly fewer lung tumours than *Huwe1*^*+/+*^ wild type (WT) mice, with a 68% reduction in mean tumour number (61 vs 15 for WT and KO, respectively) (Fig. 2B, Di). In line with this, a previous study demonstrated that *Huwe1* is required for *EGFR*-induced lung tumorigenesis in vivo and further attributed this to the degradation of p53 following HUWE1 ubiquitination [13].

To determine whether an additional, p53-independent mechanism exists, we incorporated a *Tp53*^*Flox/Flox*^ allele into our model to generate the KP GEMM, in which Ad-Cre delivery resulted in the expression of mutant *Kras* G12D and simultaneous knockout of *Tp53* by excision of essential exons 2-10 (Fig. S4A). Knockout of p53 resulted in a more aggressive tumorigenesis phenotype, with substantial tumour burden observed throughout the distal lung after 4-6 months. Again, mice with concomitant *Huwe1* depletion showed a significant and dramatic reduction in lung tumour formation with 86% reduction in mean tumour number (28 vs 4 for WT and KO, respectively) (Fig. 2C, Dii), further demonstrating the importance of *Huwe1* in the development of mutant *Kras*-induced lung tumours. Crucially, this highlighted a previously unreported, p53-independent contribution by HUWE1 in LUAD tumorigenesis.

We edited the *Kras* G12D allele by CRISPR to encode the G12C variant (KCP GEMM), which is more commonly detected in human LUAD, accounting for ~30% of all *KRAS*-mutant cases [37]. Here, *Huwe1* depletion again resulted in a significant reduction in tumour formation with an 86% reduction in mean tumour number (14 vs 2 for WT and KO, respectively) (Fig. S4B, C), providing further evidence for the oncogenic requirement for HUWE1 in *Kras*-induced LUAD development in vivo.

As an independent model for *Kras*-induced murine lung tumorigenesis, we utilised urethane chemical carcinogenesis, which preferentially induces *Kras* Q61 hotspot mutations resulting in distal lung tumour formation [38]. This also exhibits a mutational burden more closely resembling human LUAD compared to *Kras* Cre-inducible models [39]. The multiplicity of urethane-induced lung tumour formation is more typical of human disease in comparison to Cre-inducible models, which result in several tumours throughout the distal lung. *Huwe1*^*+/+*^ and *Huwe1*^*Flox/Flox or Flox/y*^ mice were initially administered Ad-Cre, followed by 10 weekly doses of intraperitoneal urethane **(**Fig. 2E**)**. Whilst control *Huwe1*^*+/+*^ WT mice consistently exhibited tumour formation, mice with initial *Huwe1* depletion showed a reduction in tumour incidence (WT: 100% incidence, KO: 50% incidence) and multiplicity (WT: 75% >1 tumour, KO: 17% >1 tumour) (Fig. 2F, G). Additionally, urethane-induced lung tumours were typically smaller in *Huwe1* KO mice compared to *Huwe1* WT mice (Fig. S4D). This agrees with our previous findings and highlights that HUWE1 is required for both direct oncogene-induced and carcinogen-induced murine LUAD development.

HUWE1 IHC staining following an optimised protocol for murine samples (Fig. S5A–C) revealed that all tumours tested that formed in both WT and KO mice in the KP GEMM retained HUWE1 expression at a similar stain intensity (Fig. S5D, E). Based on this IHC staining, we speculate that the small number of tumours that form in *Huwe1* KO mice could be a result of ‘escaper’ events caused by inefficient Cre-lox recombination at the *Huwe1* locus – which would further indicate the importance of HUWE1 in the formation of *Kras*-induced lung tumours in vivo.

To assess whether *Huwe1* depletion is specifically a vulnerability of transformed LUAD cells, and control for any impact of *Huwe1* loss in the healthy, normal cell of origin - alveolar type 2 (AT2) cells - we utilised a tamoxifen-inducible Cre expressed under the AT2-specific surfactant protein C promoter (*Sftpc-Cre-ERT2*) (Fig. S6A, B). Following AT2-specific depletion of *Huwe1* in healthy, adult mice we found no impact on alveolar architecture (Fig. S6C), maintenance of the live AT2 population (Fig. S6D–F), or any clinical symptoms indicating toxicity or sickness. We confirmed efficient Cre-recombination of the *Huwe1*^*Flox/Flox or Flox/y*^ locus by genomic PCR from FACS-sorted AT2 cells isolated post-mortem (Fig. S6G, H). This revealed that HUWE1 is not required for the continued function, growth, or survival of healthy AT2 cells, contrasting with its essential requirement in LUAD tumorigenesis in vivo.

Together, these data strongly support the importance of *Huwe1* in LUAD tumorigenesis across murine models incorporating different *Kras* mutant isoforms, which have been associated with diverse biology and pathogenicity [40], and through both direct oncogene-induced and carcinogen-induced models. The conserved requirement for *Huwe1* across these models highlights its critical importance in LUAD initiation and tumour development in vivo. We further reveal that this is independent of HUWE1’s known regulation of p53 stability. The dichotomy of requirement for *Huwe1* between LUAD and healthy AT2 cells supports the possibility of therapeutically targeting HUWE1 for the inhibition of LUAD growth whilst limiting any toxicity in adjacent normal tissue.

### HUWE1 depletion impairs LUAD tumour cell growth in vitro and growth of established LUAD subcutaneous tumours in vivo

**Fig. 3:**
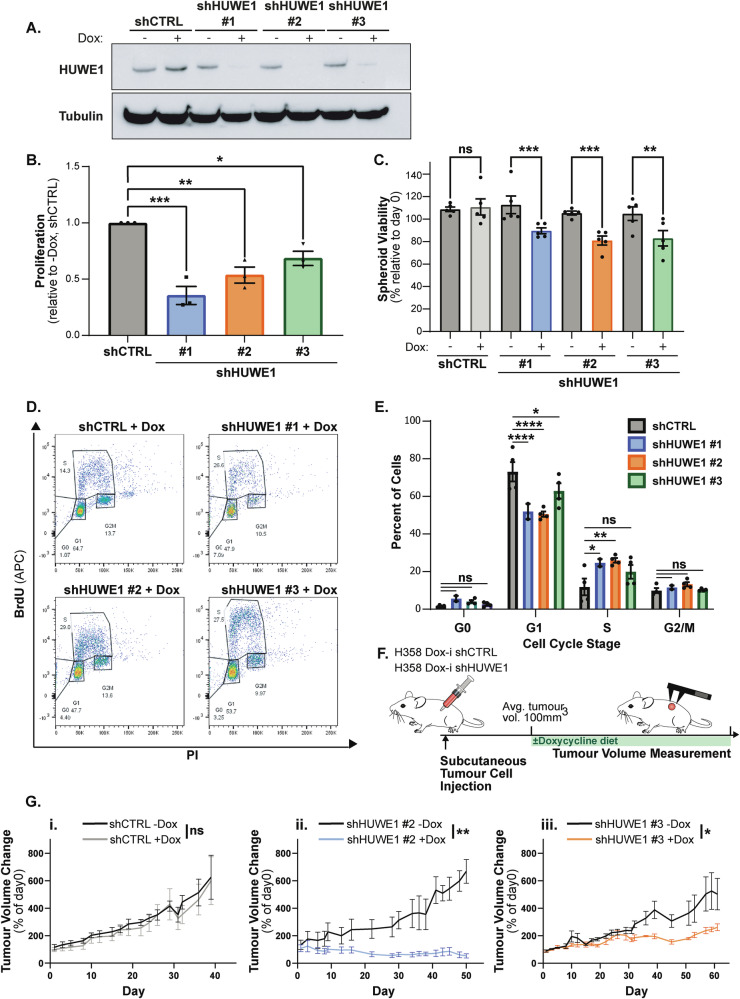
HUWE1 depletion impairs LUAD tumour cell growth in vitro and growth of established LUAD subcutaneous tumours in vivo. **A** HUWE1 protein expression in H358 cells stably expressing doxycycline-inducible control (shCTRL) or HUWE1 targeting (shHUWE1 #1-3) shRNA constructs. 10 ng ml^−1^ Doxycycline treatment for 72 hours. **B** Proliferation in 2D culture of H358 cells expressing doxycycline-inducible shHUWE1 over 10 days (treated with 10 ng ml^−1^ doxycycline) relative to matched untreated conditions and relative to shCTRL expressing cells. Proliferation is measured by cell counts.  *n* = 3 independent experiments, shown as mean ± SEM. One-way ANOVA with multiple comparisons. **C** Viability of H358 cell spheroids expressing doxycycline-inducible shHUWE1 in 3D at day 6 relative to day 0, in the absence and presence of 10 ng ml^−1^ doxycycline, as measured by 3D Cell-Titre Glow assay.  *n* = 5 independent experiments, shown as mean ± SEM. One-way ANOVA with multiple comparisons. **D** Representative flow cytometry dot-plots showing BrdU incorporation (APC -anti-BrdU) and DNA content (PI) in H358 cells expressing doxycycline-inducible shHUWE1 or shCTRL following 10 ng ml^−1^ doxycycline treatment for 10 days. **E** Quantification of cell cycle distribution from BrdU analysis as in (**D**), percent of single cells in each cell-cycle phase.  *n* = 4 independent experiments, shown as mean ± SEM. One-way ANOVA with multiple comparisons. **F** Experimental timeline for growth of subcutaneous tumour xenografts of doxycycline-inducible H358 cells expressing shHUWE1 or shCTRL in murine flanks in the absence or presence of doxycycline to assess established tumour maintenance and growth. **G** Tumour volume change over time for control and doxycycline treated groups for (i) shCTRL H358 xenograft mice (*n* = 5 vs 5), (ii) shHUWE1 #2 H358 xenograft mice (*n* = 4 vs 4), and (iii) shHUWE1 #3 H358 xenograft mice (*n* = 5 vs 5). Tumour volume change plotted relative to day 0 which denotes beginning of treatment, determined once group tumour volume averaged 100 mm^3^. Shown as mean ± SEM, two-way ANOVA with multiple comparisons.

To recapitulate our in vivo findings using a human LUAD model lacking p53, we utilised the H358 cell line, which harbours a *KRAS* G12C heterozygous mutation and a homozygous deletion of *TP53*. Depletion of HUWE1 using three independent doxycycline-inducible shRNA constructs (shHUWE1 #1-3 +Dox.) (Fig. 3A) resulted in a significant reduction in cell proliferation in 2D-culture, as well as viability of 3D-cultured spheroids compared to untreated control cells (-Dox.) and cells expressing a control shRNA (shCTRL + Dox.) (Fig. 3B, C). Bromodeoxyuridine (BrdU) incorporation revealed that HUWE1-depleted H358 cells demonstrated an atypical cell cycle distribution compared to untreated and shCTRL-expressing control cells, with a reduced population of cells in G1 and an increased percentage in S-phase (Fig. 3D, E). Taken together, these results indicate that HUWE1 depletion resulted in a prolonged or arrested S-phase, culminating in impaired cell proliferation. No induction of apoptosis was identified in H358 cells following HUWE1 depletion by AnnexinV(APC)/PI flow cytometry (Fig. S7A, B), further highlighting that the changes in cell abundance were a result of proliferative arrest rather than induction of cell death.

Having established that HUWE1 is required for lung tumorigenesis in vivo and continued proliferation of human LUAD cancer cells, we interrogated whether HUWE1 depletion could abrogate growth of an established LUAD tumour – mimicking a therapeutic intervention in the clinic. After subcutaneous implantation of doxycycline-inducible shHUWE1 or shCTRL H358 cells into athymic nude mice, tumours were allowed to develop and grow to an average size of 100 mm^3^, before mice were randomised into groups and supplemented with a normal diet or diet containing doxycycline (Fig. 3F). Whilst no difference in tumour growth was observed between doxycycline-treated and untreated shCTRL tumours (Fig. 3Gi), subcutaneous H358 tumours with HUWE1 depletion exhibited significantly reduced growth compared to their matched untreated controls (Fig. 3Gii, iii) revealing the importance of HUWE1 expression in continued LUAD tumour growth beyond tumour initiation. HUWE1 was efficiently depleted in tumours from shHUWE1 mice treated with doxycycline, compared to intact HUWE1 expression in control tumours (Fig. S7C). Doxycycline-treated shHUWE1 #2 tumours showed the greatest reduction in HUWE1 expression and exhibited the most tumour regression, suggesting that therapeutic targeting of HUWE1 could induce clinical LUAD regression.

In line with our previous in vivo findings, these data confirm that HUWE1 is required for the continued proliferation of LUAD cells in vitro, as well as the growth of established LUAD tumours in vivo – indicating that the requirement for HUWE1 is maintained beyond initial tumorigenesis.

### LUAD cells exhibit a senescent phenotype following loss of HUWE1

**Fig. 4:**
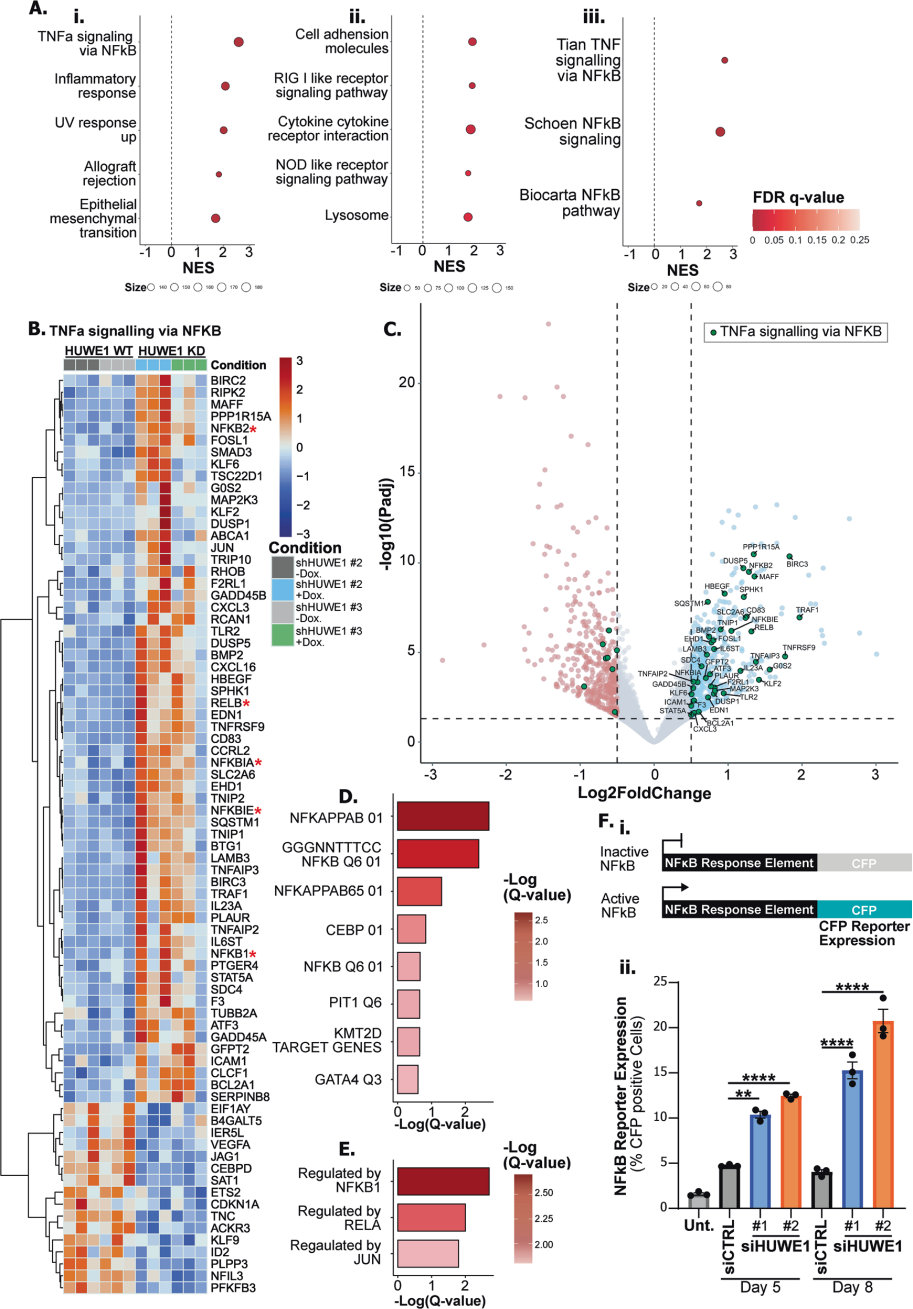
HUWE1 depletion promotes upregulation of NFκB signaling. **A** Hallmark (i), KEGG (ii), and NFκB signalling (iii) gene sets were significantly enriched (FDR q-val < 0.25) in HUWE1-depleted H358 cells. NES denotes normalised enrichment score, size denotes gene set size. **B** Heatmap depicting genes of the ‘Hallmark TNFa signalling via NFKB’ gene set, which are significantly differentially expressed between HUWE1-depleted versus control H358 cells. Core components of the NFκB pathway are depicted by an asterisk (*). **C** Volcano plot highlighting genes of the ‘Hallmark TNFa signalling via NFKB’ gene set (green) significantly differentially expressed between HUWE1-depleted versus control H358 cells. **D** Enrichment of transcription factor targets (GSEA Transcription_Factor_Targets) in the significantly upregulated (log2FC >=1) DEGs from comparison of HUWE1-depleted versus control H358 cells. **E** As in D, for enrichment of transcriptional regulatory relationships (TRRUST). **F** (i) Schematic depicting NFκB CFP fluorescent reporter construct. (ii) Quantification of NFκB transcriptional activity as percent CFP reporter positive H358 cells after 5- and 8-days transfection with control (siCTRL) or HUWE1 targeting (siHUWE1 #1/#2) constructs. *n* = 3 independent experiments, shown as mean ± SEM. One-way ANOVA with multiple comparisons.

Having identified a consistent and striking requirement for HUWE1 in the tumorigenesis and continued growth of both LUAD tumours and cancer cell lines, we performed transcriptomic analysis to investigate the mechanism of impaired cell proliferation. Following doxycycline-inducible shRNA depletion of HUWE1 (Fig. S8A), H358 cells underwent major transcriptomic reprogramming with HUWE1 expression accounting for the highest degree of transcriptomic variance (PC1) by principal component analysis (Fig. S8B, C). Differential expression analysis and GSEA highlighted that HUWE1-depleted cells exhibited increased transcription of genes associated with inflammatory signalling and NFκB pathway [41, 42] activation (Fig. 4Ai-iii). Significantly upregulated differentially expressed genes (DEGs) included key nodes of the canonical and non-canonical NFκB signalling pathway such as *NFKB1*, *NFKB2*, *RELB*, and several NFκB transcription targets (Fig. 4B, C). Transcription factor enrichment analysis and transcriptional regulatory relationship analysis further highlighted that the most upregulated genes following HUWE1 depletion were those regulated by NFκB and Rel family transcription factors (Fig. 4D, E).

To validate these findings, we transduced parental H358 cells with a retroviral CFP reporter of NFκB transcriptional activity. Upon HUWE1 depletion by transient siRNA transfection using two constructs, CFP reporter expression was increased, indicating elevated NFκB pathway activation and transcriptional activity (Fig 4Fi-ii) in concordance with our transcriptomic data.

**Fig. 5:**
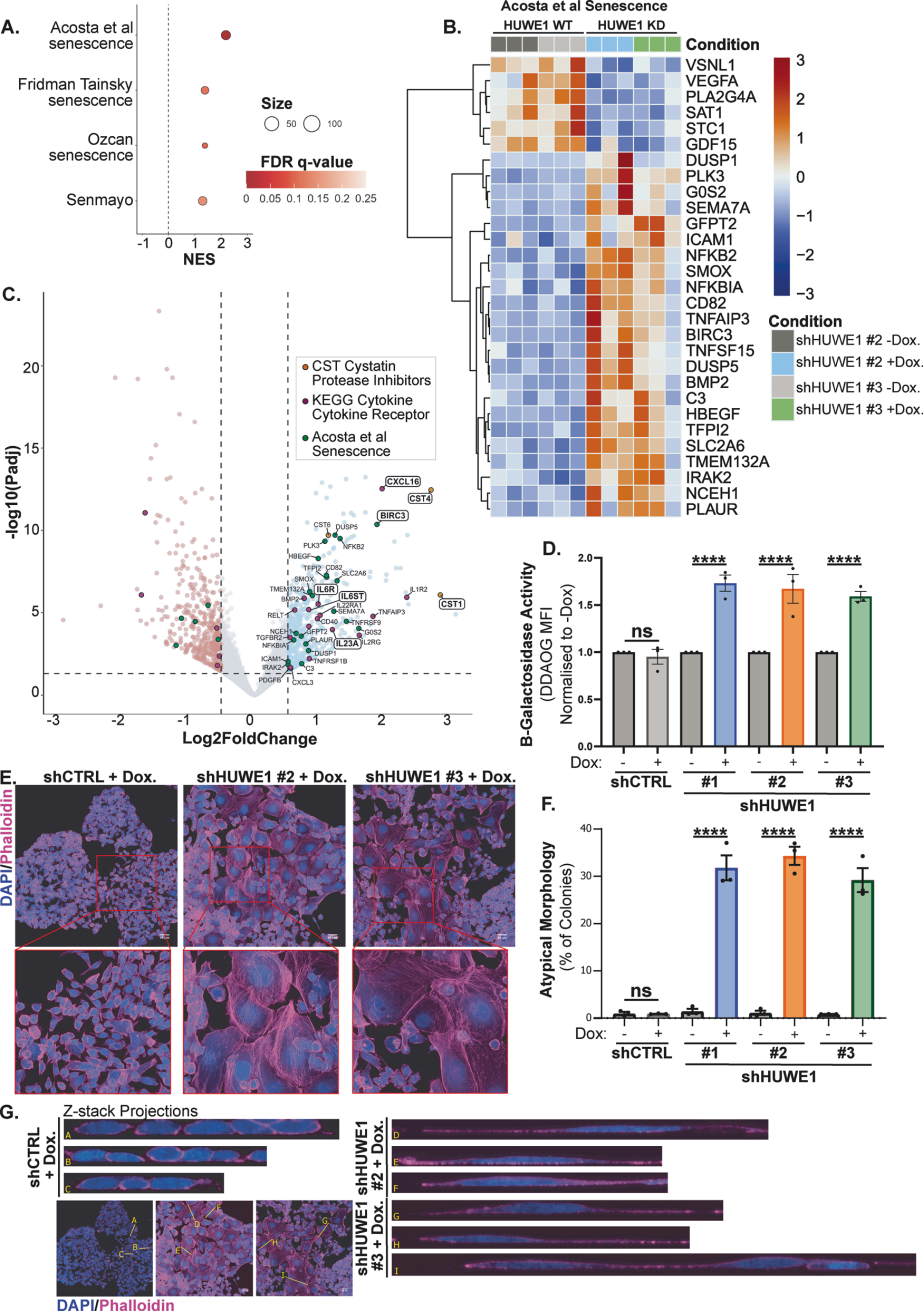
HUWE1 depletion promotes the induction of senescence. **A** Senescence gene sets significantly enriched (FDR q-val < 0.25) in HUWE1-depleted H358 cells. NES denotes normalised enrichment score, size denotes gene set size. **B** Heatmap depicting significantly differentially expressed genes upon comparison of HUWE1-depleted versus control H358 cells, which are part of the ‘Acosta et al Senescence’ gene set. **C** Volcano plot highlighting genes associated with senescence and SASP signalling significantly differentially expressed between HUWE1-depleted versus control H358 cells. ‘Acosta et al Senescence’ genes (green), ‘KEGG Cytokine - Cytokine Receptor’ genes (purple), and cystastin protease inhibitor CST genes (orange). **D** Quantification of SA-β-Galactosidase activity as measured by DDAOG median fluorescence intensity (MFI) in H358 cells expressing doxycycline-inducible shHUWE1 or shCTRL, normalised to matched untreated cells. *n* = 3 independent experiments, shown as mean ± SEM. One-way ANOVA with multiple comparisons. **E** Representative immunofluorescence images showing cell morphology in H358 cells expressing doxycycline-inducible shHUWE1 or shCTRL following treatment with 10 ng ml^−1^ doxycycline for 10 days. Phalloidin staining of F-actin in pink, DAPI staining of DNA in blue. Maximal Z-projection of Z-stack confocal images. Scale bar: 50 μm. **F** Quantification of the percent of colonies exhibiting extensive morphological atypia in H358 cells expressing doxycycline-inducible shHUWE1 or shCTRL in the absence and presence of 10 ng ml^−1^ doxycycline treatment. *n* = 3 independent experiments, shown as mean ± SEM. One-way ANOVA with multiple comparisons. Quantification performed manually by brightfield microscopy. **G** Line scans of confocal Z-stack images from **E** showing cross-section slices through H358 shRNA cells following treatment with doxycycline. All line cross-sections are at the same scale.

We also identified significantly altered expression of gene sets associated with senescence [43–46] following HUWE1 depletion (Fig. 5A). NFκB signalling is commonly activated by senescence [47, 48] and is also typically associated with the senescence-associated secretory phenotype (SASP), which culminates in expression of a range of inflammatory mediators (chemokines, cytokines, interleukins, growth factors) and receptors involved in inflammatory signalling [49, 50]. Key mediators of senescence signalling elevated following HUWE1 depletion included the anti-apoptotic regulator cIAP2 (*BIRC3*), and regulators of inflammatory SASP signalling, including core components of interleukin-6 signalling (*IL6R*, *IL6ST*), interleukin 23a (*IL23A*), and the cytokine *CXCL16* (Fig. 5B, C). Additionally, cystatin protease inhibitors *CST1* and *CST4*, which are reported markers of senescent cells [51] were two of the topmost significantly upregulated genes following HUWE1 depletion (Fig. 5C).

These transcriptomic findings are in line with the observed impairment in proliferation and atypical cell cycle distribution – both of which are classical characteristics of cells undergoing senescence [52]. Assessment of senescence-associated β-Galactosidase activity by flow cytometry analysis of the DDAOG far-red probe revealed that HUWE1 depletion resulted in a significant increase in β-Galactosidase activity in H358 cells (Fig. 5D, S9A, B) – providing further evidence that H358 cells undergo senescence following loss of HUWE1 expression.

Additionally, we observed that following HUWE1 depletion, H358 cells undergo a morphological change with the emergence of cells with an enlarged cytoplasmic area, enlarged nucleus, and signs of both increased intracellular granularity - typical of vacuolation - and increased nuclear atypia (Fig. 5E, Fig. S9C). Immunofluorescence and confocal microscopy of filamentous-actin (F-actin) and nuclear architecture revealed that these enlarged cells exhibit a reduced nuclear height and dramatically elongated cell size – typical of the large, flat cells observed during senescence [53] (Fig. 5G). We identified many F-actin filaments in HUWE1-depleted, enlarged cells, and Z-projection cross-sections revealed basal puncta of F-actin, indicating that these were basal actin stress fibres (Fig. 5E, G), which are also associated with the induction of senescence [54, 55]. Following HUWE1 depletion, we also identified increased protein expression of p21 in H358 cells in line with the induction of senescence (Fig. S9D).

Finally, we assessed the protein expression of several HUWE1 ubiquitination substrates reported from other cell contexts and identified no alteration in their expression following HUWE1 depletion in H358 cells (Fig. S9E), indicating that their dysregulation was not underlying the senescent phenotype. Pilot proteomics experiments to ascertain the mechanism underlying the role of HUWE1 in LUAD and how HUWE1 depletion results in cellular senescence revealed evidence of HUWE1’s involvement in multiple cellular stress signalling pathways. This included the regulation of DNA replication and replicative stress signalling, as well as protein quality control pathways that regulate endoplasmic reticulum (ER) stress and the unfolded protein response.

These in vitro findings corroborate our previous in vivo data by revealing that in a p53-independent model of LUAD, HUWE1 depletion results in impaired cell proliferation and the induction of senescence.

## Discussion

In this study, we identified that HUWE1 expression is upregulated in LUAD compared to normal lung tissue, and that this increase corresponded with tumour stage – suggesting the importance of HUWE1 upregulation in both initial development and malignant progression of LUAD tumours. Across multiple independent murine models, we have demonstrated that depletion of *Huwe1* resulted in a significant reduction in *Kras*-induced lung tumour development, supporting the functional importance of HUWE1 in LUAD tumorigenesis. Interestingly, this requirement for *Huwe1* is maintained across both oncogene and carcinogen-induced murine models and following introduction of different *Kras* hotspot mutations (G12D, G12C, Q61L/R), which are associated with diverse pathogenicity and incidence. It is conceivable that the requirement for HUWE1 may extend beyond *Kras*-induced LUAD tumorigenesis, which is supported by reported evidence that *Huwe1* loss abolishes tumour formation in an *EGFR-VIII* LUAD murine model [13]. Interestingly, the requirement for HUWE1 in LUAD tumorigenesis contrasts with its dispensable nature for the maintenance of the cell-of-origin of LUAD since HUWE1 depletion did not impact AT2 cell proliferation or survival in vivo. These data reveal a dichotomy of importance for HUWE1 in normal AT2 cells and transformed LUAD cells.

Whilst our findings provide compelling evidence for the importance of HUWE1 in LUAD tumorigenesis and continued LUAD cell proliferation, the molecular mechanism through which HUWE1 acts remains elusive. Murine tumorigenesis experiments with concomitant *Tp53* depletion revealed a p53-independent mechanism for HUWE1 in LUAD tumorigenesis, distinct from its previously reported ubiquitination and regulation of p53 stability [13].

We observed that HUWE1 depletion in human LUAD cells caused the induction of senescence as determined by the identification of many archetypal characteristics of senescent cells. These included impaired cell proliferation, an atypical cell cycle distribution, transcriptomic reprogramming involving activation of inflammatory SASP and NFκB signalling, emergence of morphologically atypical enlarged and flattened cells, and upregulated SA-β-Galactosidase activity. We identified some heterogeneity in the temporal induction of senescence, with not all cells becoming senescent in a uniform manner upon HUWE1 depletion. However, despite this, we observed an increase in the protein levels of p21, an established senescence marker. Surprisingly, the cell cycle aberration we identified was not the classical G1 arrest often associated with senescence. Several reports, including a study that also used H358 cells, revealed that prolonged and arrested S-phase can also contribute to the induction of senescence [56–58]. The induction of senescence – a permanently growth-arrested state – highlights a critical role for HUWE1 in the maintenance of cell cycle progression and proliferation of LUAD cells.

The expression of reported HUWE1 ubiquitination substrates did not change following HUWE1 depletion, suggesting a novel mechanism. Based on pilot proteomics experiments, we speculate that loss of HUWE1 induces sustained activation of complex intracellular stress signalling pathways, including DNA replicative stress and endoplasmic reticulum (ER) stress, which culminate in proliferative arrest and the induction of senescence. These findings are in line with previous reports that HUWE1 regulates protein quality control pathways to clear misfolded or aggregated proteins and prevent unfolded protein response signalling [59–61], and that HUWE1 ubiquitinates DNA replication machinery in the regulation of origin firing and alleviation of replicative stress [62, 63]. The direct ubiquitination substrates underlying these data remain elusive and are the subject of further investigation.

Data reported here are the first evidence that HUWE1 depletion induces activation of an NFκB-mediated transcriptional programme culminating in tumour-cell senescence. In line with these findings, it has been previously shown that HUWE1 depletion in human pulmonary artery endothelial cells results in NFκB activation via the stabilisation of RelA [64]. Intriguingly, HUWE1 has also been reported to negatively regulate NFκB signalling in some contexts, including through appending protective, branched polyubiquitination on TRAF6 [65], and via interaction with HAPSTR1 in the nucleus [66]. It is likely that these conflicting roles for the regulation of NFκB by HUWE1 are due to its context-dependent ubiquitination; the heterogeneity of HUWE1 substrate ubiquitination is highlighted extensively in the literature, where comparison of differentially expressed proteins between different cell types following HUWE1 depletion reveals considerable variation [59]. Additionally, context-dependent signalling by HUWE1 is exemplified in colorectal cancer, in which HUWE1 can both potentiate and repress Myc transcriptional activity through ubiquitination of either Miz1 or c-Myc [17, 18].

We also revealed in this study that HUWE1 depletion results in increased inflammatory SASP signalling as LUAD cells become senescent; we hypothesize that this may contribute to immunogenic clearance of senescent LUAD cells in vivo to ameliorate tumour growth. However, pilot experiments to deplete CD8+ cytotoxic T-lymphocytes in vivo followed by induction of tumour formation suggested this mechanism is not CD8+ T-cell dependent. Alternatively, another immune compartment may contribute to anti-tumour immunity following HUWE1 depletion and subsequent inflammatory signalling during induction of senescence. This will be the subject of further investigation, including determining whether HUWE1 depletion and subsequent inflammatory changes could be exploited to increase the efficacy of immunotherapy in LUAD.

Importantly, when we allowed subcutaneous human LUAD tumours to develop in the murine flank and subsequently depleted HUWE1, we identified a significant growth defect in these tumours – this highlights that the functional requirement for HUWE1 is maintained beyond LUAD initiation, and that targeting HUWE1 presents a rational therapeutic strategy for inhibiting the continued growth of established LUAD tumours. Existing small molecule inhibitors of HUWE1 suffer from poor specificity and pharmacokinetic properties [18]. Recent advances in elucidating the full, high-resolution protein structure of human HUWE1 may enable more specific, efficacious drug design [67]. Alternatively, our data support the candidacy of HUWE1 as an E3 ligase for recruitment by PROTAC or molecular glue compounds, exploiting the elevated expression of HUWE1 in LUAD as a mechanism to specifically ubiquitinate and degrade essential proteins such as mutant KRAS.

In conclusion, our study highlights a pivotal role for the E3 ubiquitin ligase HUWE1 in both the initial tumorigenesis of *Kras*-induced LUAD and the continued growth of established tumours. We provide experimental evidence that targeting HUWE1 in LUAD is a rational strategy for intervention, which should be explored further, and may promote tumour cell senescence, a reduction in tumour growth, and benefits in patient survival.

## Materials and Methods

### Antibodies and constructs

Constructs and antibodies used in this study are detailed in Table 1 and Table 2, respectively.

**Table 1:** Plasmid constructs used in this study.

Plasmid	Source	Details
LT3 GEPIR	Addgene #111177	Doxycycline-inducible shRNA expression backbone. 3^rd^ generation lentivirus.
pMDLg/pRRE	CRUK National Biomarker Centre (NBC), Manchester. (Addgene #12251)	Viral packaging plasmid
pRSV-REV	CRUK National Biomarker Centre (NBC), Manchester. (Addgene #12253)	Viral packaging plasmid
pMD2.G (VSVG)	CRUK National Biomarker Centre (NBC), Manchester. (Addgene #12259)	Viral packaging plasmid
pSIRV-NF-KB-eCFP	Addgene #118094	CFP reporter under NFκB response element. Retrovirus.

**Table 2:** Antibodies used in this study.

Antibody	Species	Vendor	Catalogue Number	Application and dilution
Anti-rabbit IgG, HRP-linked secondary	Rabbit	CST	#7064S	1:5000 (WB)
Anti-mouse IgG, HRP-linked secondary	Mouse	CST	#7076S	1:5000 (WB)
Anti-Lasu/Ureb1 (HUWE1)	Rabbit	Bethyl	#A300-486A	1:2000 (WB)
Anti-HUWE1/Mule	Mouse	Abcam	Ab70161	1:500 (Murine IHC)
Anti-HECTH9 (HUWE1)	Mouse	CST	#5695	1:50 (Human IHC)
Anti- α-Tubulin	Mouse	Sigma	T9026	1:5000 (WB)
Anti- β-Actin	Mouse	Sigma	A5441	1:10,000 (WB)
Anti-BrdU, APC-conjugated	Mouse	Biolegend	364114	5 µl per 100 µl cell sample (Flow Cytometry)
Anti-TIAM1	Sheep	R&D Systems	AF5038	1:250 (WB)
Anti-Mcl1	Rabbit	CST	#5453	1:1000 (WB)
Anti-KRAS	Rabbit	LS Bio	LS-C175665	1:1000 (WB)
Anti-cMyc	Rabbit	CST	#9402	1:1000 (WB)
Anti-p21 Waf1/cip1	Rabbit	CST	#2947	1:500 WB

### Analysis of LUAD patient datasets

TCGA_LUAD and CPTAC-3_LUAD transcriptomics and clinical datasets were accessed from the Genomic Data Commons (GDC) portal using the TCGABiolinks Bioconductor package in RStudio. Normalised gene expression values were extracted, and HUWE1 expression was plotted between normal lung and tumour samples. For matched comparisons, paired statistical analyses were performed. Normalised gene expression data for normal lung and LUAD were analysed by gene set enrichment analysis in GSEA software (Broad Institute), using default settings. A false discovery rate (FDR) q-value of <= 0.25 was considered statistically significant enrichment. A positive normalised enrichment score (NES) denotes enrichment in the LUAD tumour samples. Normalised TMT-quantitative proteomics data from CPTAC-3_LUAD patients were accessed from Gillette et al. [68]. HUWE1 expression was extracted and plotted between normal lung and tumour samples.

### Analysis of DepMap data

Dependency data for both RNAi (DEMETER2 DATA V6) and CRISPR (DepMap Public 23Q4) mediated loss-of-function screens were accessed through the DepMap portal (https://depmap.org/portal/). Data was filtered for LUAD cell lines, and known E3 ligase dependency scores were extracted and plotted by rank for comparison of E3 ligase dependency.

### HUWE1 IHC analysis of LUAD patient samples

FFPE samples from stage I (*n* = 20), II (*n* = 20), and IIIa (*n* = 13) patients were collated within a tissue micro-array (TMA) sourced via the ETOP (European Thoracic Oncology Platform) Lungscape study (REC Ref 12/LO/0235) with sample collection following patient informed consent. Samples were sourced and assessed from the Pathology Department, Wythenshawe Hospital, Manchester Foundation Trust, and were de-identified prior to use in this study. The design and construction of the TMA were in line with the ETOP Lungscape ALK-1 Pathology Protocol. In brief, two FFPE blocks were identified from each of the selected and approved patient cases that met the inclusion criteria and quality assurance parameters of the study. Two 0.6 mm cores were sampled from identified regions of the tumour for each block. A total of 4 cores of tumour tissue were included for each case. Additionally, 36 stage IV diagnostic FFPE samples, along with their clinical pathological data, were also sourced from the ChemoRes (Molecular mechanisms underlying chemotherapy resistance, therapeutic escape, efficacy, and toxicity) study (REC Ref 07/H1014/96). One full FFPE block was provided for each patient.

Serial 4 µm FFPE sections were stained for H&E and HUWE1 IHC, respectively, as detailed in **supplementary methods**. In brief: following dewaxing, antigen retrieval, and blocking, HUWE1 staining was performed with a 1:40 dilution of murine HUWE1 monoclonal antibody, clone AX8D1 (Cell Signalling #5695), overnight at 4 °C, prior to visualisation with anti-mouse polymer and DAB, and counterstaining with Haemotoxylin. FFPE sections from siCTRL and siHUWE1-transfected human LUAD cell pellets were stained alongside patient samples as specificity controls. HUWE1-stained sections were assessed blindly by a histopathologist, with review of both tumour and normal lung tissue samples. Cytoplasmic and nuclear HUWE1 staining was scored as 0 (no staining), 1 (weak staining), or 2 (moderate staining). For patients with >1 core on the TMA, a mean score was calculated. Survival analyses were performed in GraphPad Prism software with a 5-year follow-up threshold applied.

### HUWE1 survival analysis

LUAD patient overall survival and progression-free survival were stratified by median HUWE1 gene expression in multivariate analyses and Kaplan-Meier survival curves generated using the KMplotter tool (https://kmplot.com/analysis/). A cohort of *n* = 1161 LUAD patients generated by the KMPlotter tool through a combination of patients from the following studies: GSE14814, GSE68465, GSE31908, GSE29013, GSE30219, GSE19188, GSE3141, GSE31210, GSE50081, GSE37745.

### Animal experiments

Animal procedures were carried out in accordance with the Home Office Regulations (UK) and the UK Coordinating Committee on Cancer Research guidelines using approved protocols (Home Office Project license numbers: 70/8386 and PP5790814, and Cancer Research UK Manchester Institute Animal Welfare and Ethical Review Advisory Body). Mice were housed in individually vented cages in groups of 2–4 mice per cage. Caged mice were kept in an environment maintained under uniform temperature and humidity, under 12 hour light/dark cycles. Mice were monitored by weighing twice weekly. Full details of all animal procedures are provided in the **supplementary methods**. In brief: Cre-lox tumorigenesis experiments were performed by intra-tracheal/nasal administration of Adenoviral-CMV-Cre (University of Iowa viral vector core), and tumour burden was measured post-mortem by assessment of H&E-stained lung sections for all identifiable lesions. Urethane carcinogenesis was induced by once weekly intraperitoneal dosing of urethane (750 mg per kg body weight) for 10 weeks, with a rest week after 5 doses. AT2 HUWE1 depletion experiments were performed by delivery of 4 mg tamoxifen in corn oil by oral gavage for three consecutive days to *Sftpc-Cre-ERT2* mice. For LUAD tumour xenograft experiments, 2.5 × 10^6^ LUAD cells were prepared in 1:1 RPMI 10% FBS and Cultrex Basement Membrane Extract (BME) Type III per mouse, and were subcutaneously injected into the flank of athymic nude mice; tumour size was measured using digital callipers and volume was calculated by *Volume = Length/2 x width*^*2*^, animals were randomised onto normal or doxycycline diets once the average tumour volume for a group reached 100mm^3^.

### Murine lung dissociation and AT2 isolation

Full details of fresh lung dissociation and AT2 staining are provided in the **Supplementary methods**. In brief, lungs were dissected, and parenchyma minced manually with a scalpel prior to incubation at 37 °C for 90 minutes with 0.1% Collagenase-A (Sigma) and 2.5 Units/ml Dispase (Corning). After 70 mm filtration, centrifugation, and red blood cell lysis, non-specific epitope binding was blocked with Trustain FcX anti-mouse CD16/32 antibody (Biolegend) for 10 minutes on ice, followed by staining for 1 hour in the dark with FACS buffer containing BV421-CD31, BV421-CD45, APC-Epcam, and PE-MHC-II antibodies (Table 3). Subsequently, cells were washed and incubated with 1:1000 LIVE/DEAD Fixable Blue Stain Kit (ThermoFisher) for 1 hour on ice. Cells were analysed and sorted by fluorescence-activated cell sorting (FACS) using the Aria-III Cell Sorter (BD Biosciences). Viable alveolar type 2 (AT2) single cells were gated as live, CD31-, CD45-, Epcam+, MHC-II+ cells and were isolated by FACS-sorting, prior to snap freezing of subsequent pellets and storage at −80 °C.

**Table 3:** Fluorophore-conjugated antibodies were used in this study for in vivo flow cytometry analyses.

Antibody	Fluorophore	Catalogue Number	Analysis
**BV421-CD31** **anti-mouse** (clone 390)	Brilliant Violet 421	Biolegend #102423	[402 nm] 450/50
**BV421-CD45** **anti-mouse** (clone 30-F11)	Brilliant Violet 421	Biolegend #103133	[402 nm] 450/50
**APC-CD326** **Ep-CAM** **anti-mouse** (clone G8.8)	APC	Biolegend #118213	[640 nm] 670/30
**PE - MHC Class II (I-A/I-E) mouse monoclonal antibody** (clone M5/114.15.2)	PE	eBioscience #12-5321-82	[561 nm] 586/15

### AT2 cell DNA extraction and HUWE1 recombination PCR

AT2 cell pellets were thawed on ice and genomic DNA extracted using the QIAGEN DNeasy Blood and Tissue Kit as per manufacturers protocol. 75 µl PCR reactions were prepared on ice containing 50 ng genomic DNA, 200 µM dNTPs, 10% DMSO, 0.5 µM forward and reverse primers (sequence below), 1x Q5 Reaction Buffer (NEB), 1x High GC Enhancer (NEB), and 0.75 µl (1.5 Units) Q5 Hot Start High-Fidelity DNA polymerase (NEB). PCR reactions were incubated in a thermocycler with a 59 °C annealing temperature, 4-minute extension time, and for 33 cycles. PCR products were resolved in a 1% agarose gel. Bands were visualised using the BioRad GelDoc and analysed in ImageJ (FIJI).

Forward primer: 5’- GGGCCTAGTTTATCTGCTTGAA-3’

Reverse primer: 5’-GCCAGAGCTCATGTACTCACT-3’

### Cell culture

All cells initially acquired from the Cell Signalling group were frozen and identity confirmed by human cell line authentication (HCLA) services by CRUK MI Molecular Biology Core Facility (MBCF). Cells were maintained in incubators at 37 °C, 5% CO_2_ in media as detailed in Table 4. Mycoplasma screening is performed periodically by CRUK MI MBCF.

**Table 4:** Cell lines used in this study.

Cell Line	Details	Mutations	Culture Media
NCI-H358	NSCLC Cell line	*KRAS* G12C (heterozygous) *TP53* deletion (homozygous)	RPMI-1640 (Gibco), 10% tetracycline-free Foetal Bovine Serum (FBS) (Biosera), 1% Penicillin/Streptomycin solution (Gibco).
NCI-H441	NSCLC Cell line	*KRAS* G12V (heterozygous) *TP53* R153L (homozygous)	
Lenti-X HEK 239T	Lentivirus production cell line	N/A	DMEM (Gibco), 10% tetracycline-free FBS (Biosera), 1% Penicillin/Streptomycin solution (Gibco)
Phoenix-GP HEK 293T	Retrovirus production cell line	N/A	
Murine tumour cell line	Murine lung tumour cell line, developed from KP GEMM by the Cell Signalling group	*Kras* G12D (heterozygous) *Trp53* deletion (homozygous)	

### Stable cell line generation

Doxycycline-inducible shRNA cell lines were generated by ligating dsDNA oligos encoding HUWE1 or control Renilla luciferase targeting shRNA sequences (Table 5) into XhoI/EcoRI digested LT3-GEPIR plasmid backbone, followed by 3rd generation lentiviral production in Lenti-X HEK 293 T cells, and viral transduction of LUAD cells followed by puromycin selection, as detailed in full in **Supplementary methods**. Doxycycline inducible shRNA cells were pre-seeded -/+ 10ng ml^−1^ doxycycline for 72 hours prior to experiments being seeded to ensure a consistent shRNA expression from the start of experiments. NF-KB-eCFP reporter cells were generated by production of pSIRV-NF-KB-eCFP retrovirus in Phoenix-GP HEK 293 T cells and viral transduction of LUAD cells. A responsive population was selected by administration of 1 ng ml^−1^ TNFα for 1 hour, followed by FACS sorting for high CFP expression with a BD Aria-III Cell Sorter. Sorted cells were maintained in the absence of TNFα for a 2-week washout period over which CFP expression was diminished.

**Table 5:** shRNA sequences used in this study.

shRNA Construct	Target	Sequence (5’-3’)
shCTRL	Renilla luciferase (non-targeting control)	TAGATAAGCATTATAATTCCTA
shHUWE1 #1	Human HUWE1	TTCCTCTGTACCAACAACCTGCT
shHUWE1 #2	Human HUWE1	TTAATGTTCGTAAAGAAGCTGC
shHUWE1 #3	Human HUWE1	TTTCCAGGCAGAATCAATAGTGCTA

### Transient siRNA transfection

10 nM siRNA (Table 6) reverse transfection was carried out during cell seeding using Lipofectamine RNAiMax transfection reagent (ThermoFisher), and Optimem reduced serum media (ThermoFisher) following the manufacturer’s protocol.

**Table 6:** siRNA sequences used in this study.

siRNA Construct	Target	Sequence (5’-3’)
siCTRL	Firefly luciferase (non-targeting control)	CGUACGCGGAAUACUUCGATT
siHUWE1 #1 Human	Human HUWE1	CAGAUAUGCAGAAACUGGUUCCAAA
siHUWE1 #2 Human	Human HUWE1	CGGAUCUGGGAACAGUACAAUUAUA
siHUWE1 #3 Human	Human HUWE1	UAGCACUAUUGAUUCUGCCUGGAAA
siHUWE1 #1 Murine	Murine HUWE1	AAGAGGACUGCAGUGUGCUAGCUUU
siHUWE1 #2 Murine	Murine HUWE1	CACACCUGCCAUGGCUGCAAGAAUU

### Transcriptomic sequencing and analysis

Doxycycline inducible shRNA cells were seeded at an equal low density −/+ 10 ng ml^−1^ doxycycline, which was replaced after 7 days. At 10 days of culture, cells were washed with PBS and RNA extracted using the RNeasy Mini kit (Qiagen) with sample homogenisation by QIAshredder (Qiagen) as per the manufacturer’s protocols. Full details of sequencing are provided in **Supplementary methods**. In brief, libraries were prepared using the QuantSeq 3’ mRNA-Seq Library Prep Kit FWD with UDI (Lexogen) on a Bravo NGS Workstation Option B (Agilent Technologies). Single read 100 bp sequencing was performed by loading 220pM pooled libraries on a NovaSeq 6000 sequencer (Illumina) with standard loading. For analysis: reads were quality-checked using FastQC and adapters were trimmed, reads were aligned to the human genome (GRCh38) using the STAR aligner, and read counts were generated using feature Counts with Ensemble GTF annotation (Homo_sapiens.GRCh38.75.gtf). Downstream analysis was performed in RStudio using PCAtools and DESeq2. Differentially expressed genes between shCTRL + doxycycline and -doxycycline comparisons were excluded from further comparisons between shHUWE1 cells. Normalised counts were exported from DESeq2 analyses, and gene set enrichment analysis for Hallmark, KEGG, Reactome, and Gene Ontology terms was performed between conditions using GSEA software (Broad Institute) and default parameters. Significantly altered DEG lists were further analysed using Metascape software for transcription factor enrichment and enrichment of transcriptionally regulated networks (TRRUST analysis). Visualisation was typically performed using the ggplot2 and pheatmap R packages.

### Brightfield microscopy, abnormal morphology quantitation

Brightfield images were taken of cells using an EVOS XL Core Imaging System (ThermoFisher). Illumination conditions were kept the same within each experiment. Images were analysed in FIJI software (ImageJ). For abnormal morphology quantitation, cells were seeded at low density in 6-well culture plates +/− 10 ng ml^−1^ doxycycline, which was replaced after 7 days. After 10 days of culture, colonies had grown from these sparsely seeded cells but had not merged, cells were washed with PBS, fixed with 3.7% paraformaldehyde in PBS for 20 minutes at room temperature, and 70% glycerol was added for storage at 4 °C. Normal and abnormal appearing colonies were manually counted by microscopic visualisation of each well, with abnormal colonies classified as having >50% area comprising cells with evidence of abnormal morphology (enlarged cytoplasm, enlarged/atypical nucleus, granularity). Only colonies of >5 cells were included. 300 colonies were counted per well, unless fewer than 300 colonies were present, in which case all colonies were counted. The percentage of atypical colonies in each well was calculated.

### F-actin staining and confocal microscopy

Doxycycline-inducible shRNA cells were grown in 6-well plates on glass coverslips for a 10-day period in the absence or presence of 10 ng ml^−1^ doxycycline as described previously. Cells were fixed with 3.7% PFA in PBS for 20 minutes at room temperature, permeabilised in 0.5% v/v Triton X-100 (Sigma) in PBS for 3 minutes, blocked for 1 hour rocking at room temperature in 1% v/v BSA in PBS, and incubated with 0.165 µM Alexa-fluor 647 Phalloidin (Molecular Probes, Invitrogen) in 150 µl 1% BSA in PBS for 1 hour in the dark to stain F-actin. Coverslips were washed and mounted using ProLong Gold Antifade Mountant (Molecular Probes, Invitrogen) containing DAPI. Cells were analysed on the LSM 880 Airyscan laser scanning confocal microscope (Zeiss). Microscope control, image acquisition, and stitching of tile scans were performed by Zen Black (Zeiss) software, with laser intensities kept the same between conditions. Images were exported into FIJI (ImageJ) software for further analysis. Signal intensity was set to the same across all images in an experiment, and maximal Z-stack projections were generated. For line-scan analyses, lines were drawn across cells of interest in Fiji, and stacks resliced to produce a cross-section image through all Z-stacks.

### NFkB reporter experiments

NFkB fluorescent reporter cells were seeded and transfected with siRNA as described above. Three additional untreated wells were seeded for control conditions. 24 hours prior to experimental endpoints, one control well was treated with 10 ng ml^−1^ TNFα (ThermoFisher), and one with 100 nM Phorbol-12-myristate-13-acetate (PMA) (Sigma) and 100 nM Ionomycin (Sigma). At experimental endpoints, all conditions were washed with PBS, harvested by trypsinisation, and fixed in 3.7% PFA in PBS for 15 minutes at room temperature. Fixed cell pellets were kept in PBS at 4 °C in the dark until flow cytometry analysis. Cells were analysed using the LSR Fortessa X-20 flow cytometer (BD). Analysis of CFP percent positivity of single cells was performed in FlowJo software. Gates were maintained across all samples in all biological repeats.

### Statistical analyses

All statistical analyses were performed using GraphPad Prism software, comparing means of biological replicates. Data plotted as mean −/+ standard error of the mean (SEM). Statistical tests, group sizes, and number of biological replicates are detailed in the respective figure legends. Typically, the t-test is used for the comparison of two conditions, and ANOVA is used for comparison of more conditions with correction for multiple comparison testing. Equivalent non-parametric tests are used upon failure of normality testing, and paired tests are performed when comparing paired data. Experiments were performed in biological replicates to ensure reproducibility. Variance accounted for in statistical testing, including correction for non-sphericity using Geisser-Greenhouse correction where appropriate.

## Supplementary information

Supplementary Material

Original Data

## Data Availability

The RNA-seq data reported in this paper have been deposited in the National Center for Biotechnology Information Gene Expression Omnibus (NCBI GEO) (http://www.ncbi.nlm.nih.gov/geo) under accession number **GSE290690**. All other data are included in the manuscript and/or supporting material.
