# Supplementary material for: The E3 ubiquitin ligase HUWE1 is required for KRAS-induced lung cancer
Source: Cell Death Dis. 2026 Apr 7;17(1):487. doi: 10.1038/s41419-026-08672-7 (PMC13187489; doi:10.1038/s41419-026-08672-7)
Supplement: Supplementary file 1 — Supplementary Material [file 41419_2026_8672_MOESM1_ESM.pdf]

The E3 ubiquitin ligase HUWE1 is required for KRAS-induced lung cancer

J. Searle[1,2,5,§], M. Menotti[1,5], W. J. McDaid[3,5], M. J. Baker[3,5], A. Chaturvedi[4,5], L. Ginn[1,5], H. Reed[1,5], M. Carter[4,5], F. Blackhall[3,4,5], C. R. Lindsay[3,4,5,*], A. Malliri[1,3,5,*,§]

[1.] Cell Signalling Group, Cancer Research UK Manchester Institute, The University of Manchester, Manchester, UK

[2.] Current address: Lungs for Living Research Centre, UCL Respiratory, Division of Medicine, University College London (UCL), London, UK

[3.] Division of Cancer Sciences, School of Medical Sciences, Faculty of Biology Medicine and Health, The University of Manchester, Manchester, UK

[4.] The Christie NHS Foundation Trust, Manchester, UK.

[5.] Cancer Research UK Lung Cancer Centre of Excellence, The University of Manchester, Manchester, UK

* Co-last authors

[§] Co-corresponding authors

Correspondence to: Prof. Angeliki Malliri, Email: angeliki.malliri@manchester.ac.uk or Dr. Joshua Searle, Email: Joshua.searle@ucl.ac.uk

Supplementary figures:

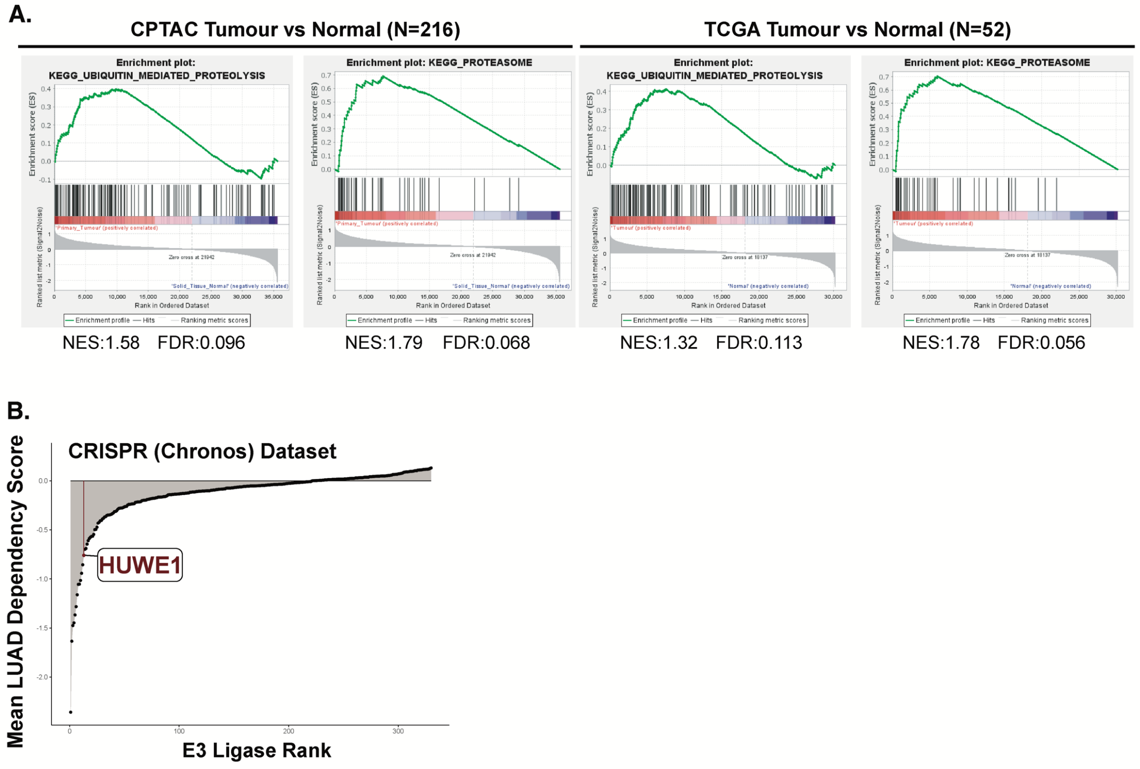

Figure S1: Components of ubiquitin signalling are upregulated in LUAD

- **A.** Enrichment plots for 'KEGG_Ubiquitin_Mediated_Proteolysis' and 'KEGG_Proteasome' gene sets from analysis in Fig 1A.
- **B.** E3 ubiquitin ligases ranked by mean dependency score for LUAD cell lines with HUWE1 highlighted, from CRISPR (Chronos) dependency data from DepMap (Broad Institute).

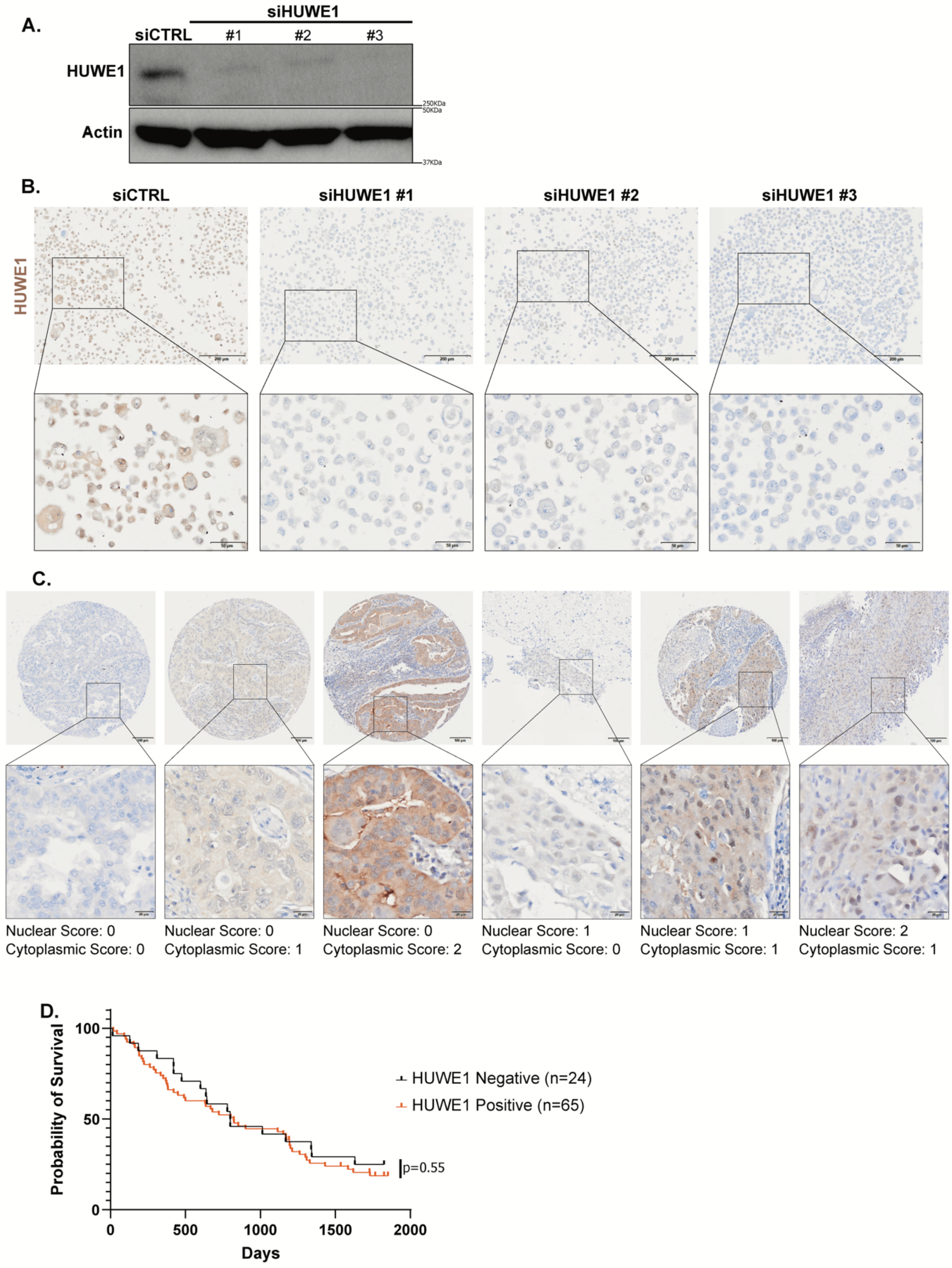

Figure S2: HUWE1 IHC reliably determines protein expression in LUAD patient samples

- **A.** HUWE1 protein expression following transient transfection with control (siCTRL) or three individual HUWE1-targeting siRNA constructs (siHUWE1 #1-3) in H441 LUAD cells.
- **B.** Representative images following optimised HUWE1 immunohistochemistry (IHC) protocol on FFPE cell pellets of H441 LUAD cells following siRNA transfection as in A. Scale bar: 200μm; inset scale bar: 50μm.
- **C.** Representative images of HUWE1 IHC stained LUAD samples and corresponding nuclear and cytoplasmic HUWE1 stain intensity scores from LUAD patient samples, scoring determined by a histopathologist. Scale bar: 100μm; inset scale bar: 20μm.
- **D.** Survival analysis of LUAD patients stratified by cytoplasmic HUWE1 expression with a 5-year follow-up period. HUWE1 negative denotes cytoplasmic score 0, n=24. HUWE1 positive denotes cytoplasmic score 1 or 2, n=65.

A.

	Stage Ia/Ib	Stage IIa/b	Stage IIIa	Stage IV
	N (%)	N (%)	N (%)	N (%)
**Cytoplasmic-/ Nuclear-**	9 (45)	7 (35)	4 (31)	3 (8)
**Cytoplasmic+**	9 (45)	12 (60)	6 (46)	13 (36)
**Nuclear+**	0	0	0	1 (3)
**Cytoplasmic+/ Nuclear+**	2 (10)	1 (5)	3 (23)	19 (53)
**Total N**	20	20	13	36

B.

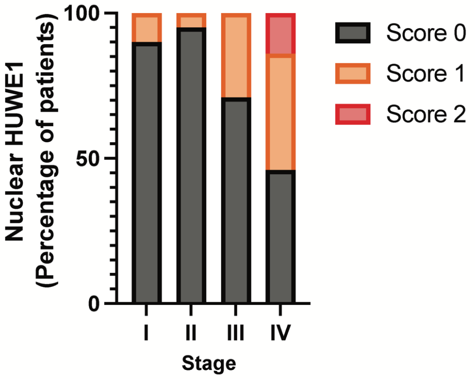

C.

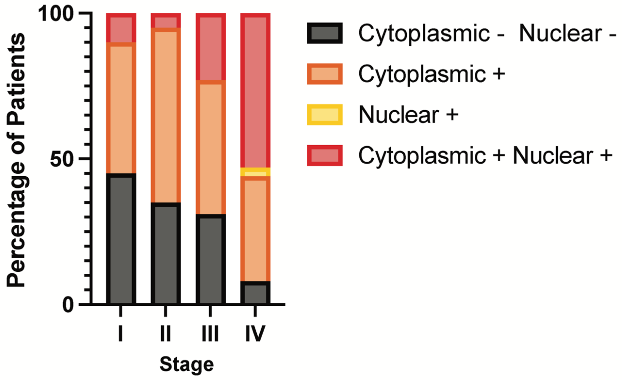

Figure S3: HUWE1 expression is upregulated by histological tumour stage in LUAD

- **A.** Table detailing numbers of LUAD patients exhibiting nuclear and cytoplasmic HUWE1 expression.
- **B.** Nuclear HUWE1 expression by histological tumour stage in LUAD patient samples.
- **C.** Cytoplasmic and nuclear HUWE1 expression by histological tumour stage in LUAD patient samples.

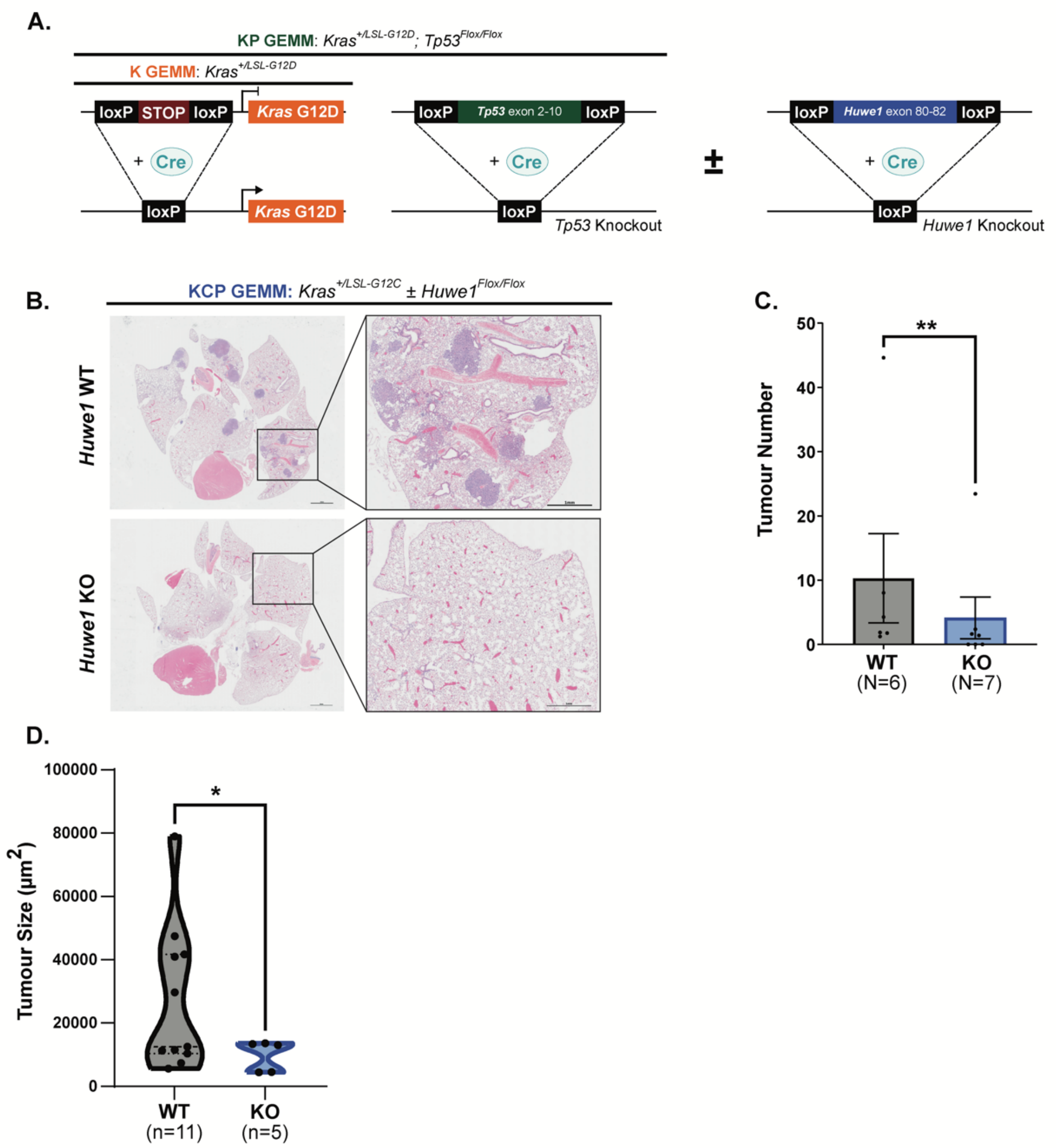

Figure S4: *Kras G12C* induced LUAD development is impaired by genetic depletion of *Huwe1 in vivo*

A. Schematic depicting Cre-lox recombination of *Kras*$^{+/LSL\text{-}Mutant}$, *Trp53*$^{Flox/Flox}$ and *Huwe1*$^{Flox/Flox\ or\ Flox/y}$ alleles.

B. Representative H&E-stained lung sections from *Huwe1*$^{+/+}$ wild type (WT) and *Huwe1*$^{Flox/Flox\ or\ Flox/y}$ knockout (KO) mice in KCP GEMM. Scale bar: 2mm; inset scale bar: 1mm.

C. Quantification of tumour number in *Huwe1* WT and KO mice in KCP GEMM. n=6 *Huwe1* WT, n=7 *Huwe1* KO. Shown as mean ± SEM, unpaired T-test with Welch's correction.

D. Quantification of tumour size for all tumours identified in *Huwe1* WT and KO mice following urethane treatment. Unpaired T-test with Welch's correction.

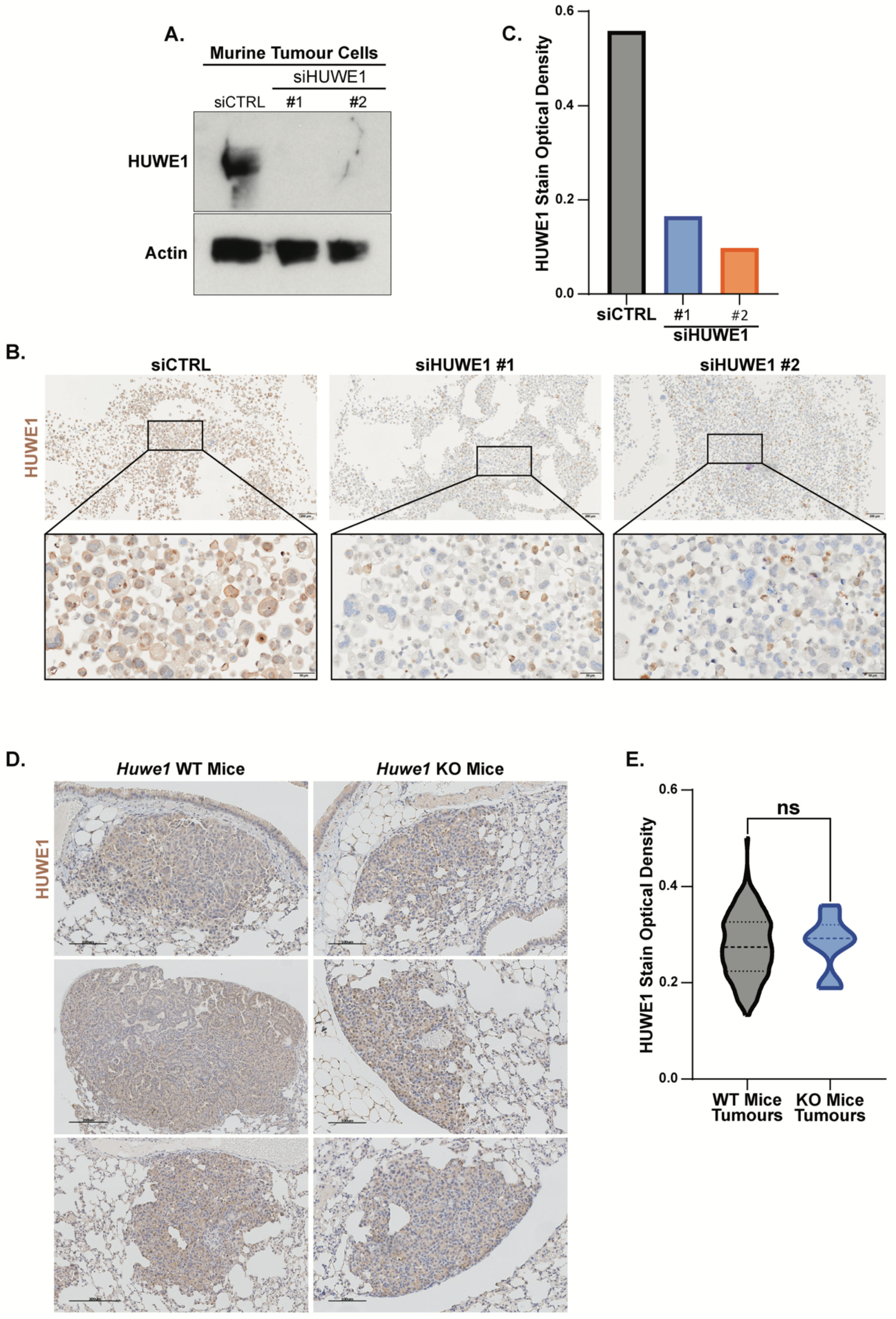

Figure S5: HUWE1 protein expression is retained in lung tumours that form in both HUWE1 WT and KO mice

A. HUWE1 protein expression in murine tumour cells (*Kras*$^{+/G12D}$, *Trp53*$^{-/-}$) transfected *in vitro* with control siRNA construct (siCTRL) or two independent HUWE1 targeting constructs (siHUWE1 #1-2).

B. Representative HUWE1 IHC staining in siCTRL and siHUWE1 transfected murine tumour cells. Scale bar: 200μm; inset scale bar: 50μm.

C. Quantification of HUWE1 stain intensity by digital image analysis of stain optical density.

D. Representative images of HUWE1 IHC staining of lung tumours in *Huwe1*$^{+/+}$ WT control and *Huwe1*$^{Flox/Flox\ or\ Flox/y}$ KO mouse samples, from KP GEMM experiments. Scale bar: 100μm (top left and all right hand images), 200μm (middle and bottom left images).

E. Quantification of HUWE1 stain intensity by digital image analysis of stain optical density in all tumours identified in *Huwe1* WT and KO mice from KP GEMM experiments. Unpaired T-test with Welch's correction.

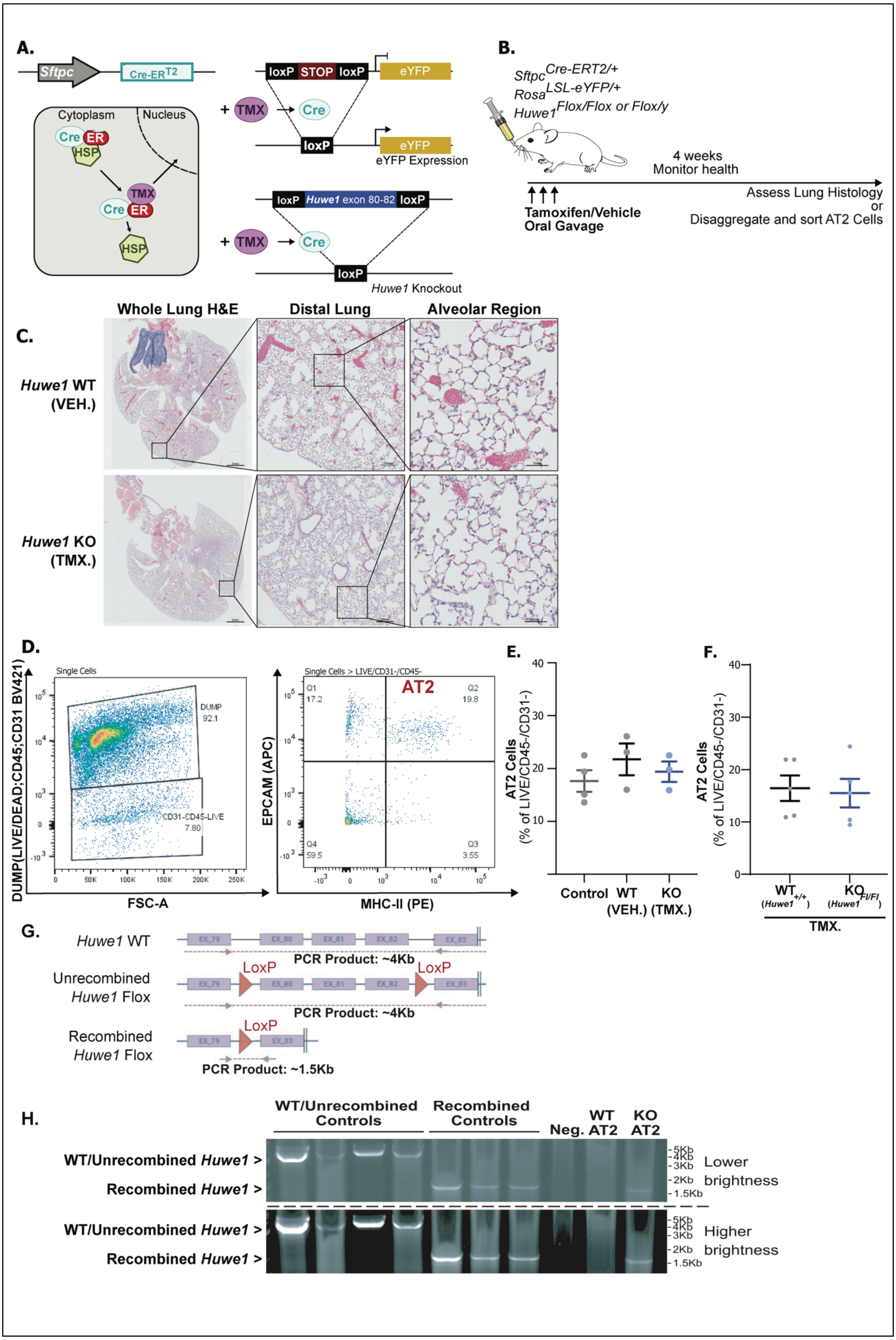
A.
Sftpc
Cre-ERT2
Cytoplasm
Nucleus
Cre ER HSP
TMX
loxP STOP loxP eYFP
Cre
loxP eYFP
eYFP Expression
loxP Huwe1 exon 80-82 loxP
loxP
Huwe1 Knockout
B.
Sftpc Cre-ERT2/+
Rosa LSL-eYFP/+
Huwe1 Flox/Flox or Flox/y
4 weeks
Monitor health
Tamoxifen/Vehicle
Oral Gavage
Assess Lung Histology
or
Disaggregate and sort AT2 Cells
C.
Whole Lung H&E
Distal Lung
Alveolar Region
Huwe1 WT (VEH.)
Huwe1 KO (TMX.)
D.
Single Cells
DUMP 92.1
CD31-CD45-LIVE 7.80
DUMP(LIVE/DEAD;CD45;CD31 BV421)
FSC-A
Single Cells > LIVE/CD31-/CD45-
AT2
Q1 17.2
Q2 19.8
Q4 59.5
Q3 3.55
EPCAM (APC)
MHC-II (PE)
E.
AT2 Cells (% of LIVE/CD45-/CD31-)
Control WT KO
(VEH.) (TMX.)
F.
AT2 Cells (% of LIVE/CD45-/CD31-)
WT (Huwe1 +/+)
KO (Huwe1 Fl/Fl)
TMX.
G.
Huwe1 WT
PCR Product: ~4Kb
Unrecombined Huwe1 Flox
LoxP
PCR Product: ~4Kb
Recombined Huwe1 Flox
LoxP
PCR Product: ~1.5Kb
H.
WT/Unrecombined Controls
Recombined Controls
Neg.
WT AT2
KO AT2
WT/Unrecombined Huwe1 >
Recombined Huwe1 >
5Kb 4Kb 3Kb 2Kb 1.5Kb
Lower brightness
Higher brightness

Figure S6: *Huwe1* genetic depletion does not impact alveolar type 2 (AT2) cell maintenance in healthy adult mice

- **A.** Schematic depicting tamoxifen-inducible Cre expression (Cre-ERT2 allele) under the surfactant protein C (*Sftpc*) promoter in AT2 cells leading to excision of loxP-STOP-loxP cassette upstream of YFP reporter in the *Rosa* locus, and excision of exons 80-82 of *Huwe1*.
- **B.** Experimental timeline of *Sftpc-CreERT2* AT2 *Huwe1* depletion murine model.
- **C.** Representative H&E-stained lung sections from mice with intact AT2 *Huwe1* (*Huwe1* WT, vehicle treated) and depleted AT2 *Huwe1* (*Huwe1* KO, tamoxifen treated). Scale bar: 2mm; first inset scale bar: 200μm; second inset scale bar: 50μm.
- **D.** Representative FACS dotplot for murine AT2 cell isolation and analysis following fresh lung disaggregation. AT2 cells defined as: CD45-, CD31-, EpCAM+, MHC-II+.
- **E.** Quantification of AT2 (MHC-II+, EpCAM+) cell population as percent of live, CD45- , CD31- single cells from AT2 *Huwe1* WT and AT2 *Huwe1* KO mice (treated with vehicle and tamoxifen respectively). Control group indicates AT2 cell population determined from additional untreated mice in preliminary study. N=3 mice per group. Shown as mean $\pm$ SEM.
- **F.** Quantification of AT2 cell population (MHC-II+, EpCAM+) as percent of live, CD45-, CD31- cells from additional experiment comparing AT2 *Huwe1* WT with AT2 *Huwe1* KO mice, all tamoxifen treated. n=5 mice per group. Shown as mean $\pm$ SEM.
- **G.** Schematic depicting genomic PCR strategy at *Huwe1* flox locus to assess loxP recombination efficiency. Red triangles represent loxP sites.
- **H.** PCR of *Huwe1* flox locus from genomic DNA extracted from FACS-sorted murine AT2 cells. Control lanes from AT2 isolation from untreated mice treated with control or Ad-Cre *in vitro*; WT and KO AT2 samples from murine study following Tamoxifen treatment to deplete *Huwe1* in AT2 cells *in vivo*. Band at ~1.5Kb represents recombined *Huwe1* locus, band at ~4Kb represents WT or unrecombined *Huwe1* locus.

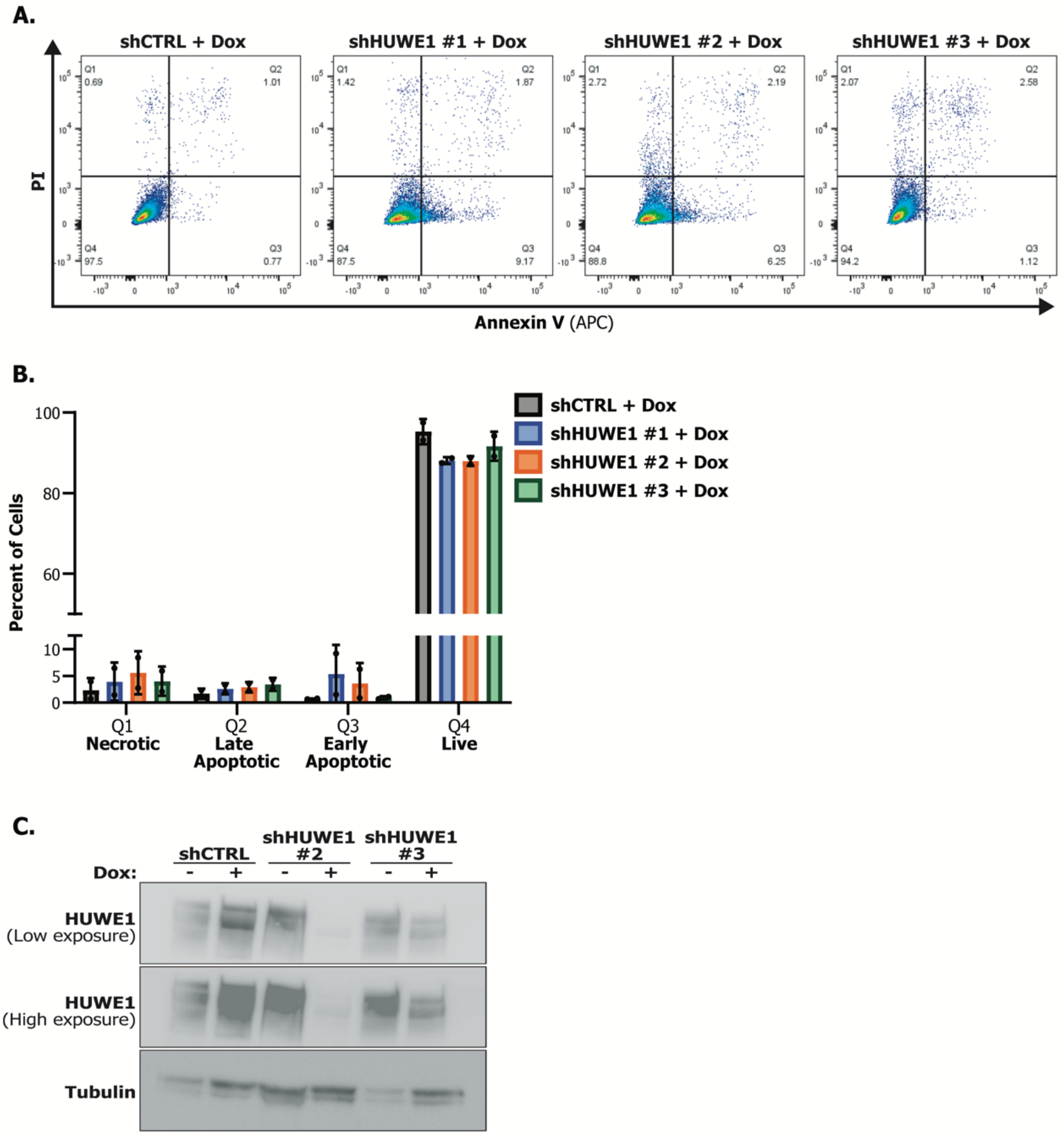

Figure S7: HUWE1 depletion does not lead to apoptosis and HUWE1 is depleted in subcutaneous shHUWE1 tumours treated with doxycycline

A. Representative flow cytometry dot-plots showing AnnexinV(APC) and PI staining to measure apoptosis in H358 cells expressing doxycycline-inducible shHUWE1 or shCTRL following treatment with 10ngml^{-1} doxycycline for 10 days.

B. Quantification of flow cytometry data as in A for each quadrant gate of single cells. N=2 biological replicate, shown as mean ± SD.

C. Representative HUWE1 protein expression in dox-inducible shRNA H358 subcutaneous xenograft tumour tissue excised and lysed post-mortem, for control (shCTRL) and HUWE1 targeting (shHUWE1 #2-3) tumours excised from mice supplemented with control or doxycycline containing diet.

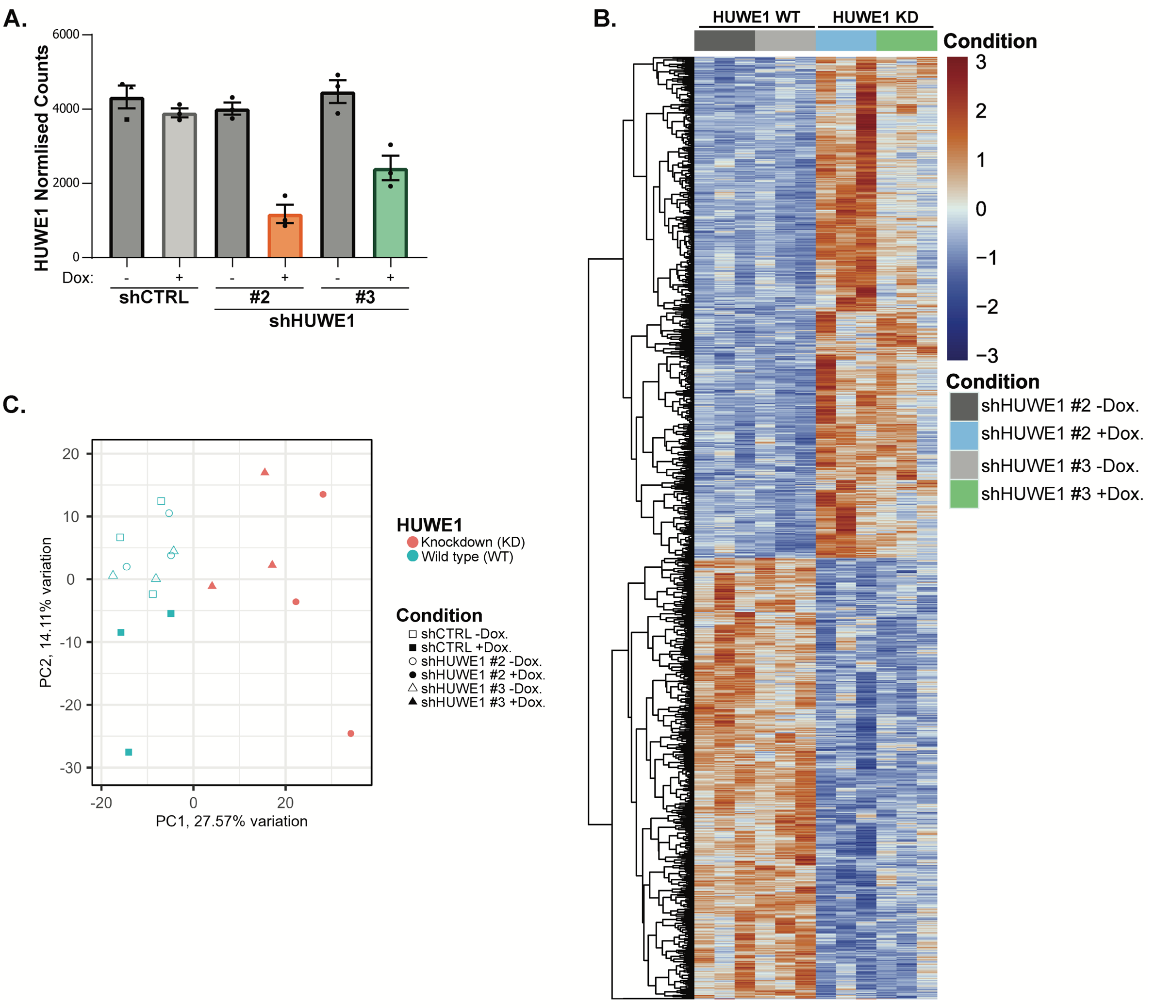

Figure S8: HUWE1 depletion induces transcriptional reprogramming in H385s cells

- **A.** Normalised counts for HUWE1 RNA transcript in H358 cells expressing doxycycline-inducible shHUWE1 or shCTRL in the absence and presence of 10ngml^{-1} doxycycline. N=3 biological repeats. Shown as mean ± SEM.
- **B.** Heatmap depicting all significant differentially expressed genes (DEGs) comparing HUWE1 depletion (shHUWE1 #2/3 + dox.) versus untreated samples (shHUWE1 #2/3 – dox.).
- **C.** Principal component analysis (PCA) showing all bulk transcriptomic samples coloured by experimental condition.

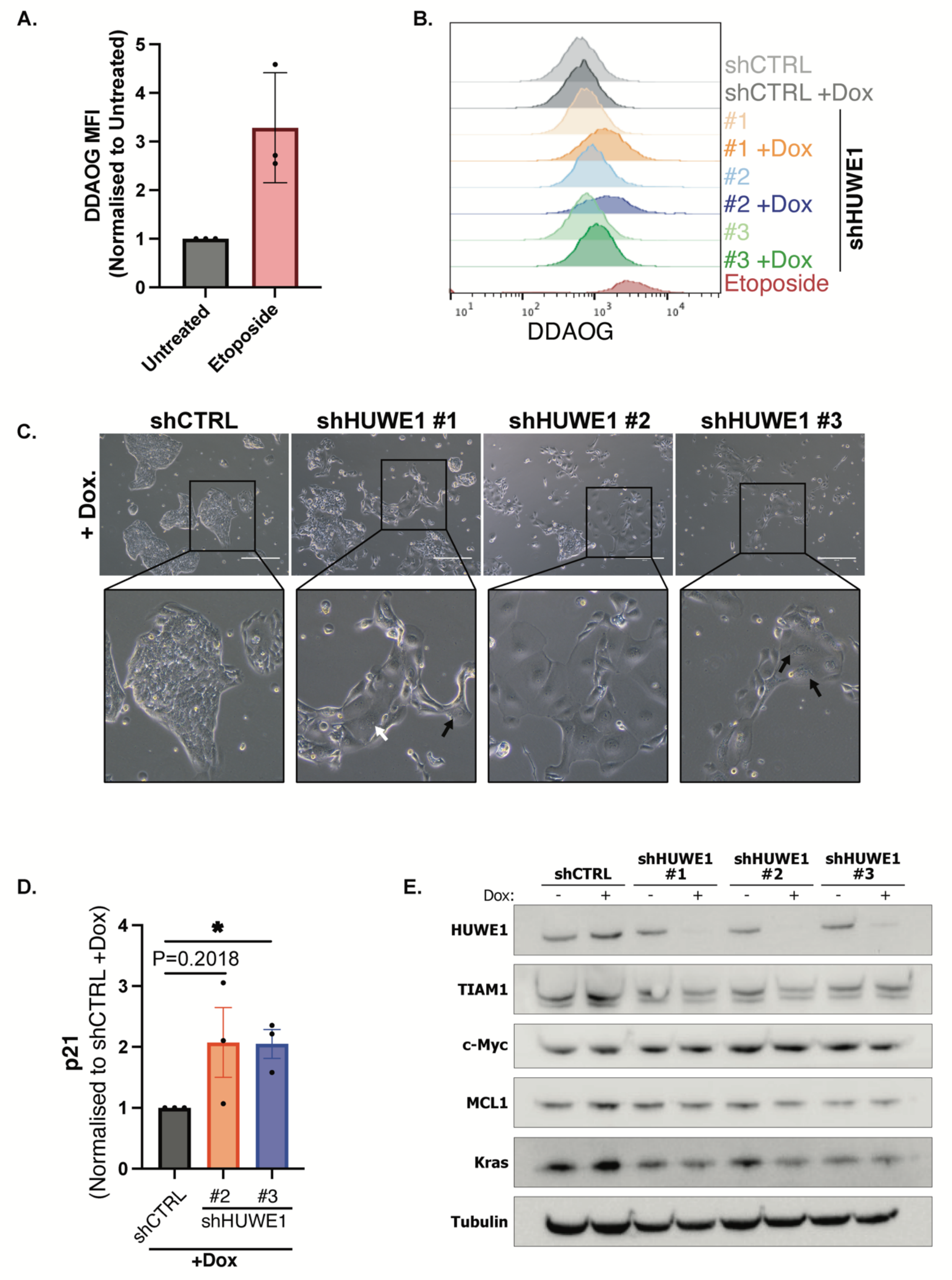

Figure S9: HUWE1 depletion promotes emergence of senescent cells

A. Quantification of DDAOG median fluorescence intensity (MFI) in untreated H358 cells and those treated with etoposide. N=3 independent experiments, shown as mean ± SEM.

B. Representative histogram of DDAOG fluorescence in H358 cells expressing doxycycline-inducible shCTRL and shHUWE1 following treatment with 10ngml^{-1} doxycycline for 10 days, or following treatment with Etoposide.

C. Representative brightfield images with high magnification insets of H358 cells expressing doxycycline-inducible shHUWE1 or shCTRL following treatment with 10ngml^{-1} doxycycline for 10 days, with evidence of extensive granulation (black arrows) and multinuclearity (white arrow). Scale bar: 400μm.

D. P21 protein expression quantified by densitometry from western blot analysis following of H358 cells expressing doxycycline-inducible shCTRL or shHUWE1 infollowing treatment with 10ngml^{-1} doxycycline for 10 days, normalised to cell number and shCTRL +Dox condition, N=3 independent experiments, shown as mean -+ SEM, One-way ANOVA with multiple comparisons.

E. Protein expression of reported HUWE1 ubiquitination substrates in H358 cells expressing doxycycline-inducible shHUWE1 or shCTRL following treatment with 10ngml^{-1} doxycycline for 10 days.

Supplementary methods:

Human HUWE1 IHC protocol:

Two serial 4µm sections were cut from FFPE patient samples and TMA blocks, mounted onto positively charged slides and dried overnight at 37°C. One section was subjected to H&E staining for detection of tumour cells, and the serial section subjected to HUWE1 IHC staining. For H&E, samples were then stained with haematoxylin and eosin (H&E) on the Leica Autostainer XL and coverslipped. For HUWE1, sections were stained using a mouse HUWE1 monoclonal antibody, clone AX8D1 (Cell Signalling #5695). Sections were dewaxed in xylene and rehydrated through an alcohol series to water. Heat antigen retrieval was applied using a digital pressure cooker (BioCare Decloaker) at 110°C for 15 minutes in citrate buffer pH 6.0 (Dako S2369). Once cooled in running water and washed in tris-buffered saline with 0.05% Tween-20 (TBS/T) (VWR), endogenous peroxidase activity was blocked with 0.3% hydrogen peroxide (VWR) for 15 minutes. Following further washing in TBS/T, sections were incubated with 10% casein (Vector SP5020) for 30 minutes to block non-specific binding. Sections were incubated with HUWE1 antibody at 1:40 dilution over-night at 4°C. Following primary antibody incubation, sections were washed in TBS/T and incubated with a peroxidase-conjugated anti-mouse polymer (Dako Envision K4001) for 30 minutes. Sections were washed in TBS/T before stain visualisation with the peroxidase chromogen, 3,3'-Diaminobenzidine (DAB) (Dako K3468) for 5 minutes. Finally, sections were rinsed in water, counterstained with Gill's I Haematoxylin, dehydrated, cleared in xylene and permanently mounted. For staining controls H441 human LUAD cells were reverse transiently transfected with 10nM siRNA targeting a non-human control sequence or HUWE1 (**Table 6**) as described below, and 72 hours after transfection were harvested by trypsinisation, washed with Phosphate Buffered Saline (PBS) and pelleted. A small sample was lysed in RIPA lysis buffer and HUWE1 protein expression analysed by western blot, as described below. Remaining cells were fixed as pellets in 3.7% paraformaldehyde (PFA) (Sigma) in PBS for 15 minutes at room temperature, embedded in paraffin and sectioned/stained alongside human tissue sections as specificity controls. Samples were processed on a ThermoShandon Excelsior processor using a standard overnight program. The tissue was then embedded in paraffin wax. Formalin Fixed Paraffin Embedded (FFPE) sections were cut at 4µm on a Leica microtome RM2255, placed on charged slides and dried over-night at 37°C.

Animal Experiments

Cre-Lox tumorigenesis experiments:

For Cre-lox tumorigenesis experiments, *Huwe1*$^{Flox/Flox}$ mice were bred with *Kras*$^{+/LSL\text{-}G12D}$ mice, and subsequently with *Tp53*$^{Flox/Flox}$ mice. The *Kras* G12D genomic sequence was edited by CRISPR-Cas9 to encode G12C as described previously[40]. All mutant *Kras* alleles were bred as heterozygous for experimental animals, *Huwe1* KO mice were bred as homozygous (*Huwe1*$^{flox/flox}$ or *Huwe1*$^{flox/y}$). Adenoviral-CMV-Cre (University of Iowa Viral Vector Core) (Ad-Cre) was prepared in ice-cold PBS and 50µl dosed to mice by intranasal or intratracheal administration. For the K and KP GEMMs each animal received $1x10^6$ plaque-forming units (PFU) of virus, for the KCP GEMM each animal received $1x10^7$ PFU virus. Tumour development was monitored by monthly micro-CT scanning, and mice were assessed twice weekly for signs of sickness including shortness of breath.

Urethane carcinogenesis experiments:

For urethane tumorigenesis experiments, *Huwe1*$^{flox/flox\ or\ y}$ mice were initially dosed with $1x10^7$ PFU Ad-Cre. Urethane (Sigma) was prepared in PBS at a stock concentration of $100mgml^{-1}$ and mice were dosed intraperitoneally (i.p.) once weekly (q.w.) with 0.75mg Urethane per gram body weight (750mg/kg) for 5 consecutive weeks, followed by a rest week, and then an additional 5 consecutive weeks. Mice were maintained on a heat pad and closely monitored during recovery due to the anaesthetic properties of urethane, which causes mice to lie torpid for up to 6 hours. Any mice showing signs of non-recovery after this period or loss of response upon stimulation were culled by a schedule 1 method of euthanasia. Mice were maintained for a period of 25 weeks from initial urethane dose prior to experimental endpoint.

AT2 HUWE1 depletion experiments:

To generate the *Sftpc-CreERT2 Huwe1*$^{flox/flox}$ line, cryopreserved sperm carrying *Sftpc*$^{tm1(cre/ERT2)Blh}$ and *Gt(ROSA)26Sor*$^{tm1(EYFP)Cos}$ alleles was obtained from Ilaria Malanchi's Tumour-Host interaction Laboratory (The Francis Crick Institute) and used to *in vitro* fertilise oocytes with a *Huwe1*$^{flox/flox}$ genotype. In brief, oocytes were collected from superovulated *Huwe1*$^{flox/flox}$ female mice and *in vitro* fertilised by the EMMA Infrafrontier protocol. 2-cell stage embryos were surgically implanted into the oviducts of CD-1 pseudopregnant mice. For tamoxifen inducible AT2 cell *Huwe1* depletion experiments, 100μl of $40mgml^{-1}$ tamoxifen in corn oil was administered by oral gavage (4mg per mouse) once daily for three consecutive days. Control mice receiving vehicle were dosed with corn oil only.

Subcutaneous xenograft experiments:

For subcutaneous injection of human LUAD cells, $2.5x10^5$ cells were prepared in 1:1 RPMI 10% FBS and Cultrex Basement Membrane Extract (BME) Type III, prior to injection of 100μl into the flank of athymic nude mice. Subcutaneous tumour width and length were measured by digital callipers thrice weekly and tumour volume calculated by $Volume = Length/2 \times width^2$. Once average tumour volume for a group reached $100mm^3$, mice were randomly assigned into groups for either normal diet or doxycycline containing diet.

Animal experiments and statistical methods

Group sizes for mouse experiments were determined with a statistician based on power calculations incorporating expected effect size and variance from previous publications using these models from ourselves and other groups and from pilot experiments, accounting for 80% power and a significance level of 0.05. Both male and female mice were included, and all mice were minimum 8 weeks of age prior to experimental use. Any mice showing clinical signs of illness prior to experimental use or treatment unrelated illness during experiments were excluded. Genetically modified mouse model experiments were not randomised as comparisons were made between genotypes. For xenograft experiments, once each group reached average tumour volume of $100mm^3$ cages were randomised onto -/+doxycycline-containing diets by computer generated randomisation, with starting tumour volumes balanced between groups at time of allocation. All mouse experiments involving Cre-recombination of engineered alleles were

performed using C57/BL6 strain mice, tumour xenograft experiments were performed using athymic nude mice. Technicians taking tumour volume measurements were blinded to groups.

Murine tissue processing:

All animals were sacrificed by methods according to the Schedule I regulation under the Animals (Scientific Procedure) Act 1986. Tissue samples for fixation and subsequent histological analyses were dissected and immediately placed in 4% buffered formalin. Lungs were re-inflated post-mortem by injection of formalin down the trachea, which was subsequently tied off with thread to ensure efficient fixation of lung tissue and displacement of air. Fresh tissue samples for subsequent protein or DNA extraction were snap frozen and stored at -80°C. Fresh lung samples for disaggregation and cell analysis were re-inflated with HBSS (ThermoFisher) and stored on ice in HBSS until processing.

Murine Lung Histological Analyses:

Following fixation for 24hr samples were processed, embedded in paraffin, and 5µm sections cut by a microtome. Sections were stained with haematoxylin and eosin on the Autostainer XL (Leica). For murine HUWE1 staining, dewaxing and heat induced epitope retrieval of slides was automated on the Bond RX (Leica), using Epitope Retrieval Solution 1 (ER1) (Leica) for 20 minutes at 98°C. Using the Bond Refine Kit (Leica) endogenous peroxidase was blocked using 3% hydrogen peroxide in TBS/T for 10 minutes and blocked with 10% casein in TBS/T. HUWE1 antibody (abcam, #70161) diluted 1:500 in Bond Antibody Diluent (Leica) was applied for 30 minutes and detected using Bond Refine kit. Slides were washed in running water and rehydrated and coverslipped with Pertex solution. For staining controls murine LUAD tumour cells isolated from a *Kras*$^{+/G12D}$ *Tp53*$^{Flox/Flox}$ mouse were reverse transiently transfected with 10nM siRNA targeting a non-targeting control sequence or *Huwe1* (**Table 6**) as described below, and 72 hours after transfection were harvested by trypsinisation, washed with Phosphate Buffered Saline (PBS) and pelleted. A small sample was lysed in RIPA lysis buffer and HUWE1 protein expression analysed by western blot, as described below. Remaining cells were fixed as pellets in 3.7% paraformaldehyde (PFA) (Sigma) in PBS for 15 minutes at room temperature, before FFPE embedding and sectioning/staining alongside murine lung sections as specificity controls. Lung tumours were manually counted from scanned H&E-stained sections and annotated in HALO (Indica) or QuPath v5.0 software. Tumours were identified by visual appearance with dense epithelial cells typically forming round or ovoid structure in the alveolar region, with enlarged nuclei.

Murine lung dissociation and AT2 isolation

Lungs were dissected and lung tissue minced manually with a scalpel prior to incubation at 37°C for 90 minutes in tissue digestion buffer (3% FBS (BioSera), 0.5mM EDTA (Gibco), in PBS), with 0.1% Collagenase-A (Sigma) and 2.5 Units/ml Dispase (Corning). Samples were gently inverted every 15 minutes to ensure thorough tissue digestion. Samples were filtered through a 70µm filter followed by wash-through of the filter with 5ml tissue wash buffer (0.5M EDTA in PBS). After centrifugation (350 rcf, 5 minutes) red blood cells were lysed (eBioscience) for 3 minutes. Cells were resuspended in cell staining buffer (1% FBS in PBS), 40µm filtered, and counted. Non-specific epitope binding by CD16 and CD32 was blocked by incubation for 10 minutes on ice with

Trustain FcX anti-mouse CD16/32 antibody (Biolegend), followed by staining for 1 hour in the dark with staining buffer containing BV421-CD31, BV421-CD45, APC-Epcam, and PE-MHC-II antibodies **(Table 3)**. After washing with cell staining buffer, cells were incubated on ice for 30 minutes with 1:1000 dilution of LIVE/DEAD Fixable Blue Stain Kit (ThermoFisher) in cell staining buffer. Following sample resuspension, single cells were analysed and sorted by fluorescence activated cell sorting (FACS) using the Aria-III Cell Sorter (BD Biosciences). Single stained UltraComp compensation beads and an unstained control sample were used for calculation of spectral compensation. Viable alveolar type 2 (AT2) single cells were gated as live, CD31-, CD4-, Epcam+, MHC-II+ cells and were isolated by FACS-sorting, prior to snap freezing of subsequent pellets and storage at -80°C.

Subcutaneous tumour tissue protein extraction

Frozen tumour and organ tissue was dissected into ~1mm^3 pieces on dry ice using a scalpel, and ~10 pieces were added to a 2ml tube containing Lysing Matrix A and a ceramic bead (MP Biomedicals) prior to tissue lysis by three one-minute cycles at an oscillation frequency of 50 Hz in the TissueLyser LT (Qiagen). Samples were clarified by centrifugation (15000 rcf, 15 minutes) and supernatant was collected. Protein quantitation by Bradford assay and western blotting was performed as described below.

Stable cell line generation:

dsDNA oligomers encoding shRNA targeting HUWE1 or a non-human Renilla luciferase control sequence detailed in **Table 5** were ligated into the XhoI/EcoRI digested LT3-GEPIR doxycycline inducible shRNA expression plasmid backbone. 5x10^6 Lenti-X HEK 293T cells were seeded into 10cm tissue culture dishes, and after 24 hours transfected using Fugene HD transfection reagent (Promega) as per manufacturers guidelines with 3.5µg respective doxycycline-inducible shRNA construct and third generation packaging plasmids (2.2µg pMDL, 1.2µg pMD2.G, 0.8µg pRSV-Rev). After 24 hours the media was replaced and 24 hours later lentivirus was harvested and 0.45µm filtered. H358 LUAD cells were seeded in 50% fresh media, 50% lentivirus supernatant, with 10µg/ml polybrene transfection reagent (Sigma). After 24 hours fresh media containing 5µg/ml Puromycin (Sigma) selection was added.

5x10^6 Phoenix-GP HEK 293T cells were seeded into 10cm tissue culture dishes, and after 24 hours transfected using Fugene HD transfection reagent (Promega) as per manufacturers guidelines with the 3µg pSIRV-NF-KB-eCFP and 3µg pMD2.G (VSVG) plasmid. After 24 hours the media was replaced with RPMI. 24 hours later media containing retrovirus was harvested, 0.45µm filtered. H358 LUAD cells were seeded in 50% fresh media, 50% retrovirus supernatant, with 10µg/ml polybrene transfection reagent (Sigma). After 24 hours fresh media was added. A responsive cell population with efficient reporter expression was selected by administration of 1ngml^{-1} TNFα for 1 hour, followed by FACS cell sorting for high CFP expressing cells on a BD Aria-III Cell Sorter. Sorted cells were subsequently maintained in culture in absence of TNFα for a 2-week washout period over which CFP expression was diminished.

Protein extraction and western blotting:

Protein extraction was performed by lysis of PBS-washed cell pellets in Laemmli buffer (BioRad) followed by heating at 90°C for 10 minutes, or by RIPA lysis buffer (ThermoFisher) containing 1:1000 PhosStop (Roche) and 1:1000 protease inhibitor cocktail (Sigma) on ice for ten minutes, followed by clarification by centrifugation at 21,000xg for 10 minutes. RIPA lysates were quantified by Bradford assay (Pierce) for equalisation as per manufacturer's protocol, Laemmli lysates were equalised by volume as equal cell numbers were lysed in a specific volume. Equalised RIPA samples were loaded in 1x NuPAGE sample reducing agent (LifeTech) and NuPAGE LDS-sample buffer (LifeTech) after heating at 90°C for 5 minutes. Equalised samples were run on 4-12% bis-tris NuPAGE gels (Invitrogen), followed by wet transfer to Immobilon-P PVDF membrane (Millipore), incubation for 1 hour in blocking buffer (5% w/v milk (Marvel) in PBS), washing in PBS/T (0.1% v/v Tween-20 in PBS), incubation with primary antibodies overnight at 4°C **(Table 2**), washing in PBS/T, incubation with secondary HRP-conjugated antibodies at 1:500 in blocking buffer, washing in PBS/T, and detection by ECL chemiluminescent substrate (PerkinElmer) and visualisation using a ChemiDoc (BioRad) or using x-ray film (GE Healthcare) exposure in a dark room, processed in a Curix60 film developer (AFGA). Developed X-ray films were scanned using an Epson perfection 3200 photo scanner at 800 dpi resolution. These images, and ChemiDoc files were analysed using Image Studio Lite V5.2 software (LI-COR). Pixel values for protein bands were determined and exported to excel, prior to normalisation against housekeeping gene loading controls. Raw uncropped blots are shown in **supplementary material**.

Proliferation, apoptosis and cell cycle distribution assays:

Prior to all doxycycline-inducible shRNA experiments, cells were pre-seeded for 72 hours in RPMI media containing 5% FBS in the presence or absence of 10ngml^{-1} doxycycline to ensure efficient shRNA expression and HUWE1 depletion from the beginning of the assay. For all *in vitro* assays using doxycycline-inducible shRNA H358s cells were maintained in RPMI containing 5% FBS for the durations of the experiment.

For 3D-spheroid cell viability assays cells were seeded in ultra-low attachment (ULA) U-bottom 96-well plates (Corning) and centrifuged (150 rcf, 5 min) and spheroids allowed to form for 24 hours. Cell-titer glow 3D (Promega) was added as per manufacturer's protocol and chemiluminescence measured using a Glo-max plate reader (Promega) and day 0 and at experiment endpoint. Viability readings were normalised to day 0, and to untreated control conditions. All measurements were made in technical triplicate, and experiments in biological triplicate. 2D cell proliferation experiments were performed by seeding cells at a low confluence +/- 10ngml^{-1} doxycycline, which was replaced at day 7. At experimental endpoint day 10, cells were trypsinised and cell counts performed. Cell counts were normalised to initial seeding density, and then to untreated control conditions. All measurements were made in technical triplicate and experiments in biological triplicate.

BrdU experiments were performed by seeding cells at a low density in 6-well plates +/- 10ngml^{-1} doxycycline, which was replaced at day 7. At day 10 cells were incubated with 100nM BrdU (Sigma) for 2 hours, washed with PBS and harvested by trypsinsation, permeabilised by dropwise addition of ice-cold 70% ethanol whilst agitating, and incubated at 4°C overnight. Samples were

incubated with 2M hydrochloric acid (ThermoFisher), 0.5% v/v Triton-X100 (Sigma) for 30 minutes, in 0.1M sodium tetraborate (Sigma) for 2 minutes, and washed in 1% v/v BSA (Sigma) in PBS. Clls were stained with APC-conjugated anti-BrdU antibody (**Table 2**) in 0.5% v/v Tween-20, 1% v/v BSA in PBS for 1 hour at room temperature in the dark, washed, and incubated with 200ul Fxcycle PI/RNase staining solution (Molecular Probes) for 20min in the dark. Single cell resuspended cells were analysed using the LSR Fortessa X-20 flow cytometer (BD Biosciences), and data analysed using FlowJo software.

For apoptosis analysis cells were seeded at low density in 6-well plates +/- 10ngml^{-1} doxycycline, which was replaced at day 7. At day 10 media was harvested containing any dead cells, and cells were detached using trypsin then recombined with their matched media and dead cells. AnnexinV(APC) and PI staining were used to identify apoptotic cells as per the manufacturers reccomendations (ThermoFisher 88-8007-72). In brief, cells were washed, stained with APC conjugated AnnexinV, washed in AnnexinV binding buffer, and subsequently stained with PI prior to analysis on an LSR Fortessa X-20 flow cytometer (BD Bioscience). Single cells were gated based on AnnexinV-APC and PI positivity to identify live, early apoptotic, late apoptotic and necrotic cells, analysis performed using FlowJo software.

DDAOG SA-β-Galactosidase analysis:

For DDAOG SA-β-Galactosidase analysis cells were seeded at low density in 6 well plates -/+ 10ngml^{-1} doxycycline which was replaced at day 7. At day 10 cells were washed in PBS, harvested by trypsinisation, washed twice in PBS. Cells were incubated for 60 minutes (37°C, shaking, no CO2, in dark) in 10ugml^{-1} DDAO Galactosidase stock solution (Invitrogen), washed three times in PBS, washed three times in cold 0.5% BSA and analysed using an Attune NxT flow cytometer (Invitrogen) with excitation/emission of DDAOG at 645/660nm. Single cells were gated and median fluorescence intensity of DDAOG signal analysis performed in FlowJo software. For positive control condition, H358 shCTRL cells were treated with 1μM etoposide for 1 week prior to analysis.

Transcriptomics sequencing and analysis:

Total RNA was quantified by Qubit RNA HS Assay (Invitrogen) and quality checked by Bioanlyzer using the RNA 6000 Pico Kit (Agilent Technologies). Indexed RNA-Seq libraries were prepared using the QuantSeq 3' mRNA-Seq Library Prep Kit FWD with UDI (Lexogen) on a Bravo NGS Workstation Option B (Agilent Technologies). An input of 500ng of total RNA was used with 15 cycles of amplification. Library quality was checked on a Fragment Analyzer using the HS NGS Fragment Kit (Agilent Technologies). Libraries were normalised based on a low output run on a MiSeq sequencer (Illumina) and a pool of the libraries quantified by qPCR using the KAPA Library Quantification Kit for Illumina (Roche). Single read 100bp sequencing was performed by loading 220pM pooled libraries on a NovaSeq 6000 sequencer (Illumina) with standard loading.

RNA-Seq reads were quality-checked using FastQC v 0.11.17 software and trimming adapters were performed. Then the reads were aligned in single-end mode to the human genome assembly (GRCh38) using the STAR aligner v 2.5.1b with default parameters. Mapped data were converted to gene-level integer read counts (expression) using feature Counts and the Ensemble GTF annotation (Homo_sapiens.GRCh38.75.gtf). Data analysis of read counts was performed using open-source R packages in RStudio software. Principle component analysis was performed

using the PCATools Bioconductor package with default settings. Differential gene expression analysis was performed using the DESeq2 Bioconductor package for comparison of counts initially between shCTRL samples in the absence and presence of doxycycline treatment, and subsequently between shHUWE1 #2 and #3 cell lines between the presence and absence of doxycycline treatment. DEGs identified in the shCTRL comparison were filtered out from further comparisons. For DESeq2 analyses differential expression was evaluated comparing the gene level integer read count data between samples, and P-values were adjusted using the Benjamini and Hochberg approach for controlling the false discovery rate. Genes with an adjusted P-value <=0.05 (FDR <= 0.05) were considered significantly differentially expressed. Normalised counts were exported from DESeq2 analyses and gene set enrichment analysis for Hallmark, KEGG, Reactome and Gene Ontology terms was performed between phenotypes using GSEA software (Broad Institute) and default parameters. Gene sets withs a false discovery rate (FDR) q-value <= 0.25 were considered significantly enriched. Significantly altered DEG lists were further analysed using Metascape software for transcription factor enrichment and enrichment of transcriptionally regulated networks (TRRUST analysis). Visualisation was typically performed using ggplot2 and pheatmap R packages.
