# Supplementary figures and images for: The E3 ubiquitin ligase HUWE1 is required for KRAS-induced lung cancer
Source: Cell Death Dis. 2026 Apr 7;17(1):487. doi: 10.1038/s41419-026-08672-7 (PMC13187489; doi:10.1038/s41419-026-08672-7)

Uncropped western blots:

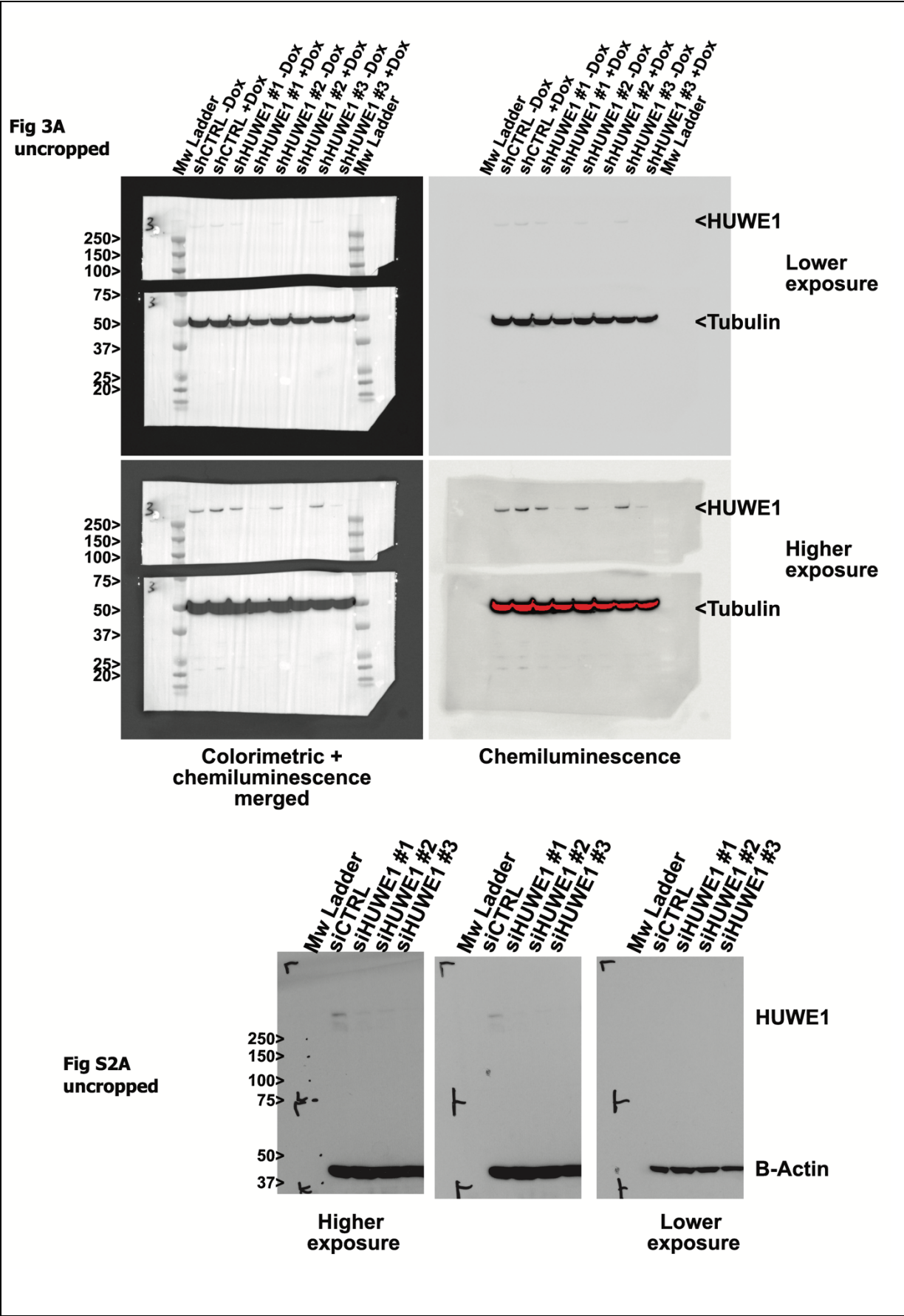

**Fig S5A**  
uncropped

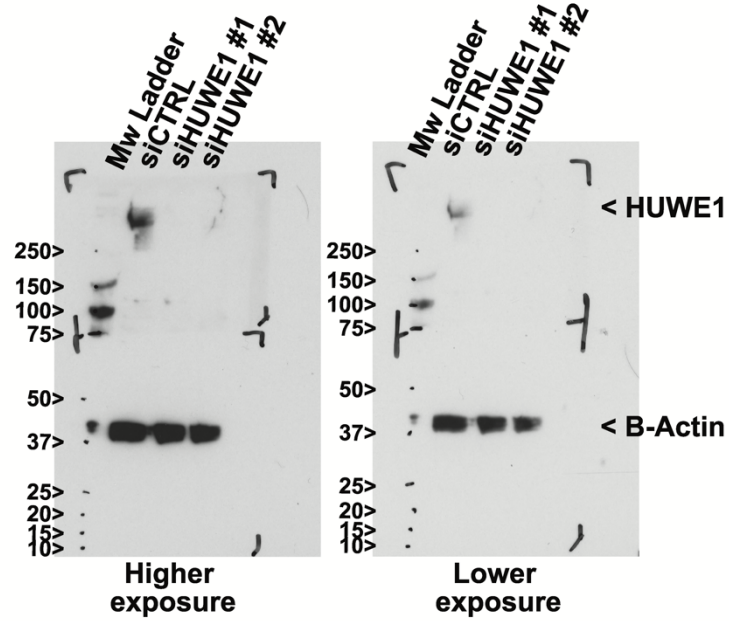

**Fig S7C**  
uncropped

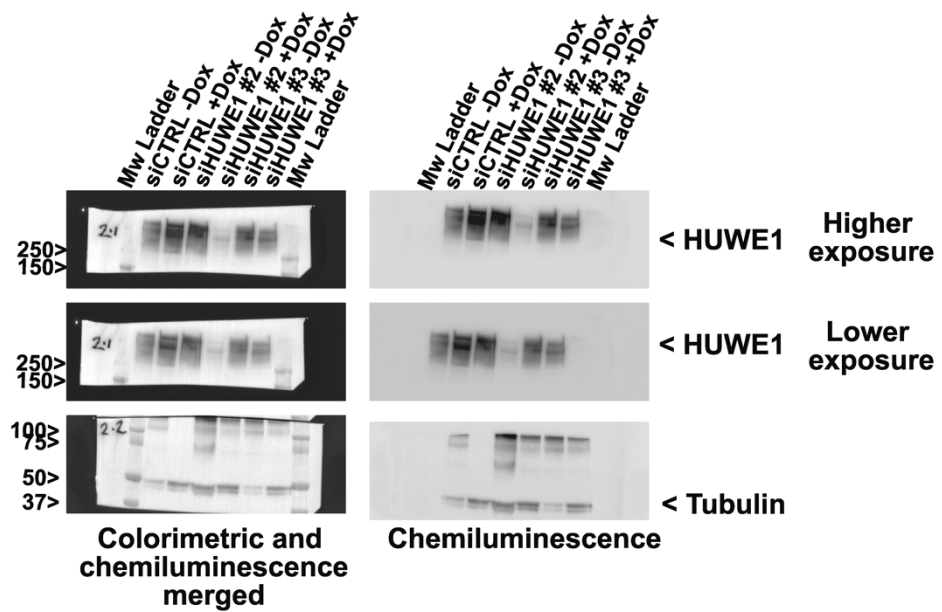

**Fig S9E**  
**uncropped**

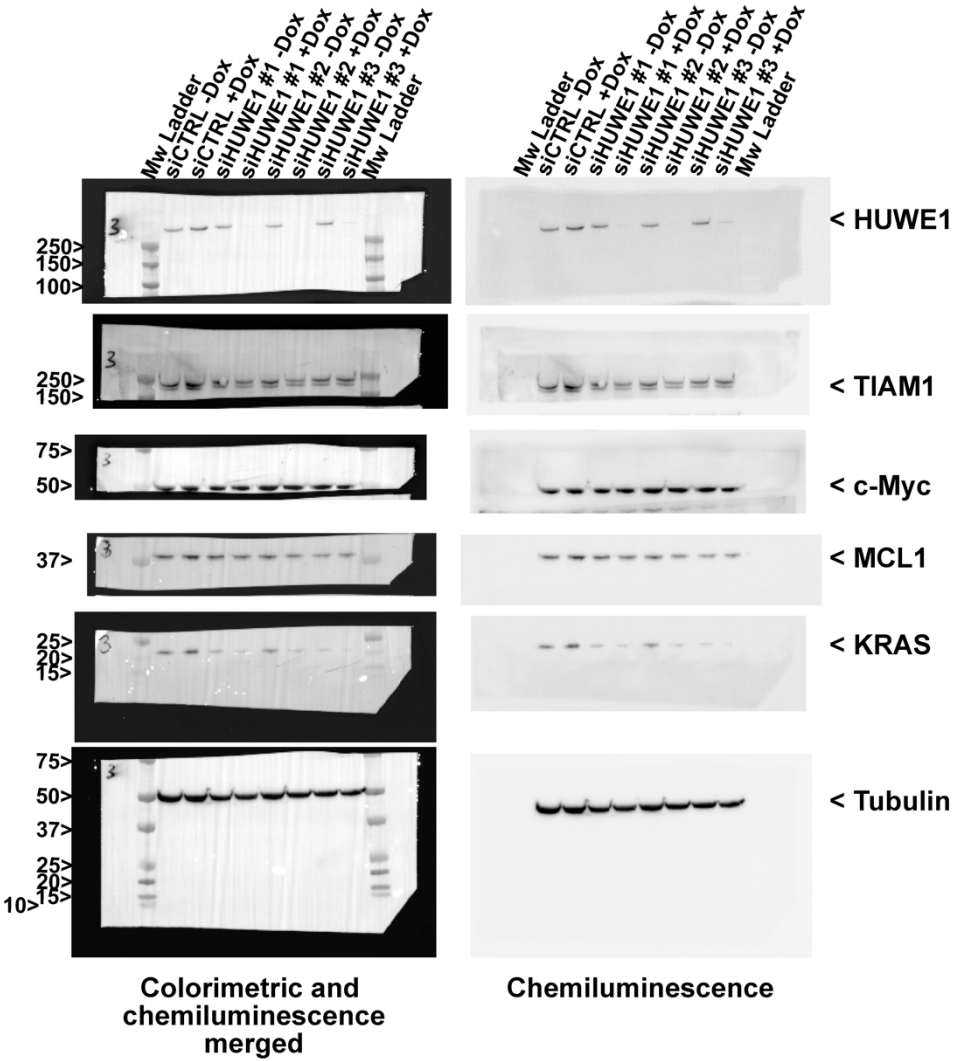

Supplement: Supplementary file 2 — Original Data [file 41419_2026_8672_MOESM2_ESM.pdf]
